# Prognostic hub gene CBX2 drives a cancer stem cell-like phenotype in HCC revealed by multi-omics and multi-cohorts

**DOI:** 10.18632/aging.205173

**Published:** 2023-11-17

**Authors:** Qingren Meng, Qian Zhou, Xi Chen, Jun Chen

**Affiliations:** 1 National Clinical Research Center for Infectious Diseases, The Third People’s Hospital of Shenzhen, The Second Affiliated Hospital of Southern University of Science and Technology, Shenzhen 518000, Guangdong, China; 2 School of Medicine, Southern University of Science and Technology, Shenzhen 518100, Guangdong, China; 3 International Cancer Center, Shenzhen University Medical School, Shenzhen 518100, Guangdong, China; 4 Shenzhen Key Laboratory of Gene Regulation and Systems Biology, Department of Biology, School of Life Sciences, Southern University of Science and Technology, Shenzhen 518100, Guangdong, China

**Keywords:** HCC, CBX2, CEP55, regulatory network, cell cycle

## Abstract

Hepatocellular carcinoma (HCC) is a malignant tumor with a high prevalence and fatality rate. CBX2 has been demonstrated to impact the development and advancement of various cancers, albeit it has received limited attention in relation to HCC. In this study, CBX2 and CEP55 were screened out with the refined triple regulatory networks constructed by total RNA-seq datasets (TCGA-LIHC, GSE140845) and a robust prognostic model. Aberrantly higher expression levels of *CBX2* and *CEP55* in HCC may be caused by CNV alterations, promoter hypo-methylation, open chromatin accessibility, and greater active marks such as H3K4me3, H3K4me1, and H3K27ac. Functionally, *CBX2*, which was highly correlated with CD44, shaped a cancer stem cell-like phenotype by positively regulating cell-cycle progression, proliferation, invasion, metastasis, wound healing, and radiation resistance, revealed by combining bulk RNA-seq and scRNA-seq datasets. *CBX2* knockdown validated its role in affecting the cell cycle. Importantly, we revealed CBX2 could activate gene by cooperating with co-regulators or not rather than a recognizer of the repressive mark H3K27me3. For instance, we uncovered CBX2 bound to promoter of *CTNNB1* and *CEP55* to augment their expressions. *CBX2* showed a highly positive correlation with *CEP55* at pan-cancer level. In addition, *CBX2* and *CEP55* may enhance extracellular matrix reprograming via cancer-associated fibroblast. Surprisingly, patients with high expression of *CBX2* or *CEP55* exhibited a higher response to immunotherapy, indicating that *CBX2* and *CEP55* may be promising therapeutic targets for HCC patients.

## INTRODUCTION

Hepatocellular carcinoma (HCC) is the major cancer type of liver cancer. High mortality, high rates of recurrence, and high rates of metastasis are characteristics of hepatocellular carcinoma [1]. Transarterial chemoembolization (TACE), ablation, liver transplantation (LT), and surgical resection are all curative-intent therapies for early-stage HCC [2, 3]. However, HCC is not entirely cured by the therapies due to the characteristics [3]. Hence, it is imperative to investigate useful prognostic biomarkers and therapeutic targets to address the shortcomings in the diagnosis and treatment of HCC.

Long non-coding RNA (lncRNA), a subtype of ncRNAs whose length greater than 200nt, could competitively bind to the miRNA, a kind of single-stranded ncRNA with length 18-25 nt, to regulate the expression of target, that is, the regular network [4, 5]. In the regular network, the lncRNAs act as the competitive endogenous RNA (ceRNA), and this hypothesis was proposed from Salmena et al. [6]. So far, increasing evidence have reported that lncRNAs could serve as ceRNA to regulate the targets by binding the miRNA sponge competitively. For instance, in HCC, lncRNA *NEAT1* altered the *miR-362-3p/MIOX* axis to increase ferroptosis [7].

*DUXAP8*, *MCM3AP-AS1* and *CDKN2B-AS1* are over-expressed in HCC, could be ceRNAs through *miR-422a/PDK2, miR-194-5p/FOXA1,* and *let-7c-5p/NAP1L1* to promote HCC, respectively [8–10]. The *DUXAP8/MCM3AP-AS1/CDKN2B-AS1 miR-424-5p/CBX2/CEP55* axis, first mentioned in HCC, was discovered in this study.

CBX2, as a key member of PRC1, recognized the sites modified by the repressive mark H3K27me3 to prevent gene transcription, indicating the abnormal expression of *CBX2* in HCC could induce a majority of genes to be silenced [11].

Here, we showed *CBX2* may enhance gene expression of *CEP55* or *CTNNB1* rather than the repressive function, especially the genes related to cell cycle, suggesting a crucial role of *CBX2* for the cell cycle. Additionally, we have identified that *CBX2* expression is cooperatively augmented by *miR-425-5p*, CNV alterations, DNA hypo-methylation, and higher active mark signals. *CBX2* affected the drug sensitivity or sensitivity by regulating the cell cycle pathway. Importantly, despite having a worse prognosis and a more advanced stage or grade, patients with high levels of *CBX2* or *CEP55* expression may still benefit more from immunotherapy. Furthermore, *CBX2* or *CEP55* could potentially serve as therapeutic targets for pan-cancer.

## MATERIALS AND METHODS

### RNA-seq data preparation, processing, and differential analysis

From the TCGA database, we downloaded the expression profiles (mRNA and miRNA) and clinical data of human hepatocellular carcinoma (LIHC). 367 HCC samples and 50 paired solid tumor-normal samples, as well as 371 HCC samples and 50 paired solid tumor-normal samples, respectively, made up the available mRNA and miRNA profiles. Downloaded from the GEO database was cohort GSE140845, whose miRNA count profile included 3 HCC samples connected to HCV and 3 paired normal samples. We performed quality control checks, read mapping operations, and finally generated the mRNA counts matrix using Fastp, HISAT2, and featureCounts, respectively, for the total RNA sequencing data in GSE140845 that were downloaded from SRP231432. The count matrices for mRNA, lncRNA, and miRNA were converted into TPM (transcripts per million) matrices and CPM (count per million mapped reads) matrices, respectively. MiRBaseVersions.db packages handled the conversion of immature miRNA names into mature miRNA names.

Then, using a count matrix and the DESeq2 R packages, we identified the differential lncRNAs, miRNAs, and mRNAs between these two cohorts with |logFC| 1 and FDR 0.01. Meanwhile, we also download the RNA-seq expression profiles from ICGC (LIRI-JP, n = 231), GSE12455 (n=70), GSE138485 (n=64), GSE144269 (n=140), GSE148355 (n=69), GSE25599 (n=20), GSE55758 (n=16), GSE94660 (n=42), and the microarray expression profiles from GSE22058 (n=197), GSE25097 (n=511), to further validate the aberrant expression of *CBX2*, and *CEP55*.

From the TCGA database, we also obtained the mutation status and CNV profiles for *CBX2* and *CEP55*.

### Establishment of the initial and refined regulatory network for HCC

Initial regulatory networks. Given that lncRNA may compete with miRNA for binding sites, acting as a natural sponge to indirectly control mRNA expression. As a result, the initial regulatory network was created using the methods described below: (1) DElncRNA-DEmiRNA pairs were constructed using the lnc2base, ENCORI, and miRWalk2.0 databases; (2) DEmiRNA-DEmRNA pairs were predicted and confirmed using the miRWalk2.0, miRWalk3.0 databases, and miRNAtap packages; (3) We combined the DElncRNA-DEmiRNA pairs and the DEmiRNA-DEmRNA pairs to create the triple regulatory network under the following circumstances: (a) DElncRNA-DEmiRNA pairs had at least one supporting piece of evidence; (b) DEmiRNA-DEmRNA pairs had more than two supporting pieces of evidence; (c) up-regulated DElncRNA might down-regulate DEmiRNA to up-regulate DEmRNAs; and (d) down-regulated DElncRNA might up-regulate DEmiRNA to down-regulate DEmRNAs.

Refined regulatory network. In the initial regulatory network, the Pearson correlation coefficient (PCC) between DElncRNA and DEmRNA was calculated. The PCC 0.3 from the initial regulatory network was used to define the refined regulatory network.

In order to determine the hub regulatory network, we used the Cytoscape plug-in CytoHubba. Based on the sequences we obtained from LNCipedia, we then used lncLocator to predict the cellular location of DElncRNA. The initial regulatory network was visualized in Cytoscape, and the final regulatory network was done in R using the ggalluvial package.

### Functional enrichment analysis

With the aid of the fgsea package, we conducted Gene Set Enrichment Analysis (GSEA) using signatures from MsigDB, such as Gene Ontology (C5.GOBP, C5.GOCC, and C5.GOMF), KEGG (C2), and Hallmark (H). The statistical significance of enriched signatures was determined by the false discovery rate (FDR) adjusted P 0.05.

The DESeq2 package was used to find the differentially expressed genes related to *CBX2*/*CEP55*. The clusterProfiler package used the DEGs to investigate potential functional pathways or Gene Ontology (biological processes, molecular functions, and cellular components).

### Survival analysis, construction of prognosis model, and validation for HCC

The *CBX2*/*CEP55*-related differentially expressed genes were discovered using the DESeq2 package. The DEGs were used by the clusterProfiler package to look into potential functional pathways or Gene Ontology (cellular components, biological processes, and molecular functions).

Prognostic variables were chosen using the randomForest algorithm from the randomForestRSC package and the LASSO algorithm from the “glmnet” package. Candidates for prognostic DERNA served as the regression’s independent variable, and overall survival and overall status in TCGA-LIHC cohorts served as the response variables. Further PH testing was done on chosen prognostic RNAs. On the chosen, a multivariate Cox regression analysis was conducted and the ggforest package was used to visualize the results. RMS R package was used to confirm nomograms as well. The prognostic model’s reliability was verified both internally and externally. By randomly dividing the TCGA-LIHC into a training cohort and a validation cohort 50 times, internal validation was carried out. And for external validation, we used the ICGC-LIRI-JR expression profile. Calibration curves, the concordance index (C-index), and the time-dependent ROC curve were used to assess the prognostic model.

The patients’ risk scores were determined using the normalized expression of confirmed prognostic RNAs and their corresponding regression coefficients in accordance with the prognostic model. Using the median risk score, the patients were then divided into high-risk and low-risk groups. To investigate the prognostic value of the risk score, univariate Cox regression analysis was used. In the Multiple Cox Regression Analysis, the risk score was combined with age, gender, and TNM stages.

### Methylation and expression analysis of *CBX2* and *CEP55*

The TCGA database was used to download the Methylation array 450K profiles that correspond to the expression profiles. Other methylation profiles, including GSE44909 (n=24), GSE73003 (n=40), GSE82176 (n=19), GSE37988 (n=124), GSE55752 (n=16), and GSE113019 (n=60), were downloaded from GEO databases. The Spearman correlation coefficient was used to assess the relationship between *CBX2*/*CEP55* and their DNA methylation level. We also investigate the effect of the *CBX2*/*CEP55* methylation level on overall survival.

### ATAC-seq, ChIP-seq analysis across human and mouse tumor cell or cell lines

The TCGA database was used to download the ATAC-seq profiles of the LIHC tumor samples, and the GSE173277 database was used to download the ATAC-seq data of the normal liver. Additionally, we downloaded the ATAC-seq data for the following cell lines from GEO: GSE172053 for Hep3B, GSE180143 for HepG2, GSE184796 for HepG2, GSE139190 for HepG2, GSE184797 for Huh7, and GSE192771 for Huh7. For the ATAC-seq analysis, we used Fastp for quality control steps, HISAT2 for read mapping onto GRCh38 reference steps, Samtools for bam file sorting, Macs2 for peak calling, and bdg2bw.sh for bedgraph file conversion into bw file. Finally, the WashU Epigenome Browser was used to visualize the tracks.

Active marks H3K4me3, H3K3me1, and H3K27ac histone ChIP-seq datasets were downloaded from GSE112221 (liver tissues), GSE113879 (liver tissues), GSE76344 (HepG2), GSE184796 (HepG2), GSE92328 (97L, LM3), GSE103730 (Huh7), GSE184797 (Huh7, PLC), GSE113879 (Hep3B), GSE172053 (Hep3B), GSE208334 (PLC), GSE168178 (Hu1545). We used analysis techniques that were similar to those used for ATAC-seq datasets.

Data for CBX2 primitive ChIP-seq was downloaded from the following GSEs: GSE29611 (K562), GSE59395 (HepG2, A549, H1), and GSE34774 (293T). The analysis was conducted in the manner described above. IDR located the peaks that were shared. HOMER2 also identified the potential motifs and the clusterProfiler package was used to conduct enrichment analysis on the genes whose close peaks were located on the promoters.

As previously mentioned, additional pre-existing data referred to as *CBX2* knockout or knockdown, such as GSE54580 (AML), GSE193477 (AML), GSE112227 (Mouse bone), and GSE156413 (Mouse embryonic fibroblasts), were also gathered and analyzed.

### Immune infiltrate levels and immune checkpoint blockade therapy prediction

The immune, stromal, and TME scores were estimated using the ESTIMATE packages. The Tumor Immune Estimation Resource 2 portal (TIMER2) databases’ immune infiltration data, including CD4 T cells and CD8 T cells, were downloaded using the CIBERSORT, MCPCOUNTER, EPIC, and QUANTISEQ algorithms. Immune inhibitor genes, immune cell score, and the Spearman correlation between *CBX2*/*CEP55* were examined.

Based on the genome mutation maf files obtained from TCGA-LIHC, the tumor mutational burden (TMB) score was calculated. The Tumor Immune Dysregulation and Exclusion (TIDE) was used to predict the effectiveness of immunotherapy and indicated immune dysfunction and escape. A low TIDE score indicated good efficacy.

The CancerSEA database [12] and Thorsson 2018 Immunity [13] were used to download the functional state signatures, which included proliferation and cell cycle. Kim et al. [14] provided a gene list for radiation resistance. Montironi et al. and Li et al. provided the inflammatory signature, inflamed signature, and IFN-gamma signature used to forecast the response to ICB [15, 16]. The ssGSEA algorithm in the GSVA package was used to determine the scores for the gene sets.

### Protein array and pathway activity calculation

The reverse phase protein array (RPPA) protein expression data were downloaded from the TCPA database. The GSCA database was used to download the pathway activity scores for the cancer-related pathways TSC/mTOR, RTK, RAS/MAPK, PI3K/AKT, hormone ER, hormone AR, EMT, DNA damage response, cell cycle, and apoptosis.

### Prediction of therapeutic agents

Based on the transcriptomic profiles and drug sensitivity data in PRISM and CTRP2, we first used the oncoPredict package to train a drug sensitivity prediction model with tenfold cross-validation. The AUC value for each drug was then predicted using the fitted model and comparable transcriptomic data.

### mRNAsi index analysis

A one-class logistic regression machine learning algorithm (OCLR), which built the model using training the human stem cell data from Progenitor Cell Biology Consortium (PCBC), calculated the mRNAsi scores of the TGCA-LIHC samples.

### Single-cell transcriptome datasets accession and analysis

Human Protein Atlas (normal), GSE140228, GSE146115, GSE98638, GSE166635, GSE140228 and GSE125449 provided the scRNA datasets for HCC. The Seurat version 4 R package was used to process the scRNA datasets.

### Immunohistochemistry and immunofluorescent staining of CBX2 and CEP55

Information on the distribution of proteins in human tissue and cells was available in the Human Protein Atlas (HPA, https://www.proteinatlas.org/), a database of the human proteome. CBX2 and CEP55 immunohistochemical and immunofluorescent staining images in Supplementary Figures 5K, 11A, 11B were downloaded from HPA (https://www.<soft-enter/>proteinatlas.org/). Detailed website information is provided in the corresponding Supplementary Figure Legend.

### Statistical analysis

R and RStudio were used to perform the statistical analyses. The Fisher’s test for discrete variables was used to compare the two groups. For continuous variables, Wilcoxon rank-sum tests and Student’s t tests were employed. The survival analysis was examined using the PH test and the log-rank test. It was deemed statistically significant when the adjusted p 0.05.

### Data and code available statement

The source data and public datasets used in this manuscript are uploaded as Supplementary Materials. The code used in this project has been uploaded to https://github.com/mengqingren/ceRNA.CBX2. All data used in this study are available as mentioned in methods, and all source data are available from corresponding authors upon request.

## RESULTS

### *CBX2*/*CEP55*-center hub-refined regulatory network

**Figure 1:**
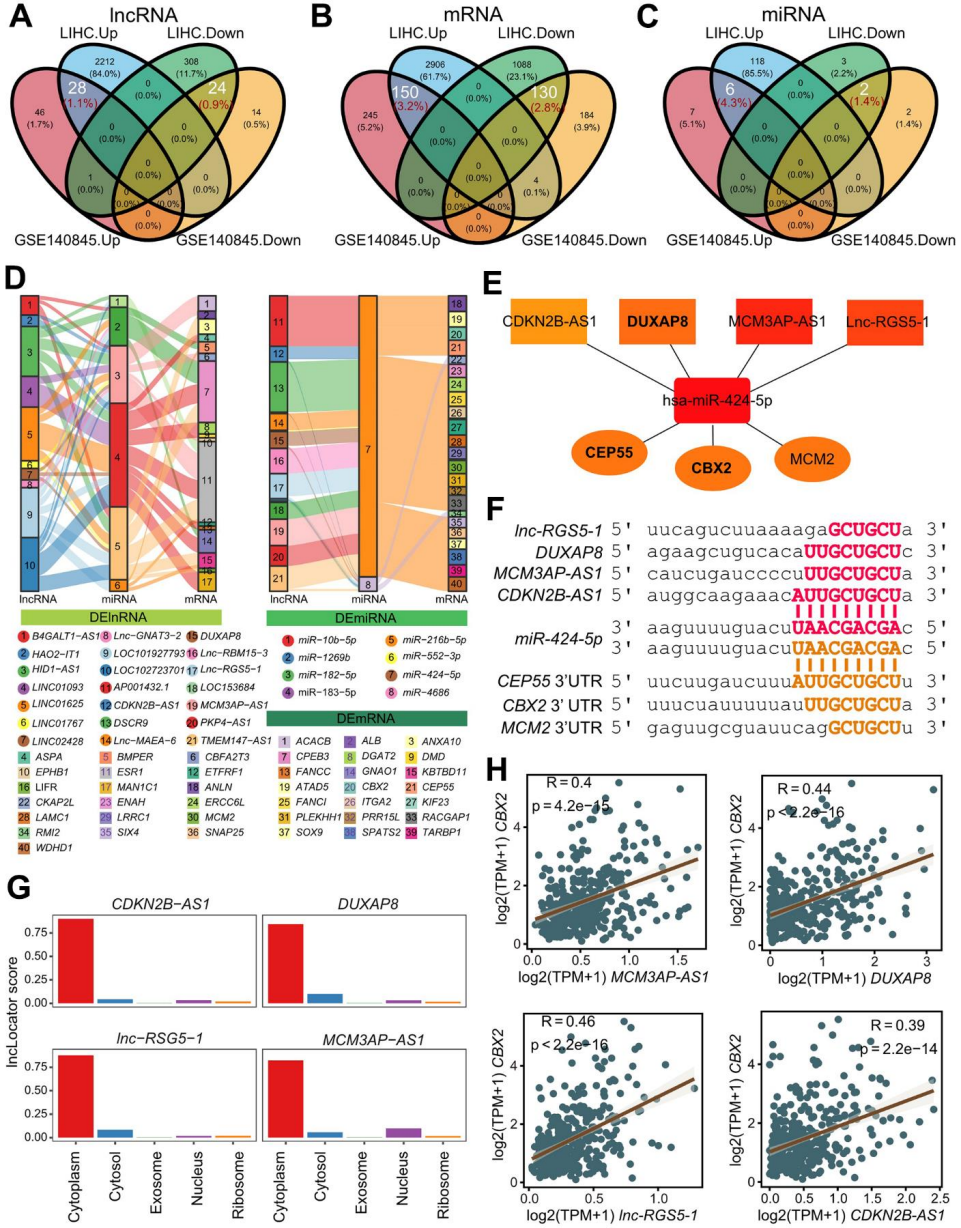
**Identification and validation of refined hubs in triple regulatory networks.** (**A**–**C**) The overlapping differential expressed genes including lncRNAs (**A**), miRNAs (**B**) and mRNAs (**C**) identified by DESeq2 (FDR:0.01, log2FC:1) with TCGA-LIHC and GSE140845. (**D**) The Sankey plot indicates the triple regulatory network based on the strategies. The thickness of lines do not make sense. (**E**) Strategy-one hub of refined triple regulatory networks determined by a Cytoscape plug-in CytoHubba. (**F**) The base pairing diagram of binding sites for the strategy-one triple regulatory network predicted by miRanda. (**G**) The predicted cellular localization for 4 lncRNAs in the strategy-one hub using lncLocator. (**H**) The Pearson’s correlation between *CBX2* and 4 lncRNAs in the strategy-one hub.

Aberrant gene expression and a poor prognosis were frequently present at the time of HCC onset and progression. To identify the regulatory networks of mRNA and ncRNAs in HCC, we collected two total RNA sequencing cohorts TCGA-LIHC (HCC=361, Adjacent=50) and GSE140845 (HCC=3, Adjacent=3), performed differential gene analysis, and found 6980 DEGs (2573 DElncRNA, 4278 DEmRNA, 129 DEmiRNA) in TCGA-LIHC and 843 DEGs (113 DElncRNA, 713 DEmRNA, 17 DEmiRNA). Finally, we established the shared genes responsible for the upregulation of 28 DElncRNA, 150 DEmRNA, and 6 DEmiRNA in HCC compared to 24 DElncRNA, 130 DEmRNA, and 2 DEmiRNA in adjacent tissue among these DEGs in two cohorts (Figure 1A and Supplementary Table 1). Heatmaps showed the top 20 genes’ expression in relation to adjacent tissues and HCC. (Supplementary Figure 1A–1F).

In order to create the refined regulatory network, we first tried to create a basic regulatory network using one of two methods: (1) up-regulated lncRNA up-regulated mRNA by down-regulating miRNA; and (2) down-regulated lncRNA down-regulated mRNA by up-regulating miRNA. By at least one database of lnc2base, ENCORI, and miRWalk2.0, the predicted lncRNA-miRNA pairs in the initial network were verified. While at least two databases - ENCORI, miRWalk2.0, miRWalk3.0, and miRNAtap -were used to confirm the miRNA-mRNA pairs. Finally, using 37 DElncRNAs (16 up-regulated, 21 down-regulated), 61 DEmRNAs (28 up-regulated, 33 down-regulated), and 8 DEmiRNAs (6 up-regulated, 2 down-regulated), the initial regulatory network was completed (Supplementary Figure 1D). We created the refined triple regulator network with 246 strategy-one routes (14 lncRNAs, 2 miRNAs, 25 mRNA), and 77 strategy-two routes (11 lncRNAs, 6 miRNAs, 17 mRNAs), by filtering out the Pearson Correlation Coefficient greater than 0.3 between lncRNA and mRNA (Figure 1D).

CytoHubba, a Cytoscape plug-in, was used to determine the hub-refined regulatory network [17]. The refined network allowed us to isolate two hub regulatory networks: Networks centered on the first strategy (*CBX2*/*CEP55*/*MCM2*) and the second strategy (ESR1) (Figure 1E and Supplementary Figure 2A). Through experimentation, it had been demonstrated that ESR1 prevented the growth and metastasis of HCC [18]. Tumor cell migration and proliferation were facilitated by *DUXAP8*, *CDKN2B-AS1*, and *MCM3AP-AS1* in HCC [8–10]. Fortunately, there was little evidence that *DUAXP8*, *CDKN2B-AS1*, and *MCM3AP-AS1* functioned as ceRNAs to control the expression of *CBX2*, *CEP55*, and *MCM2* by competitively binding has-miR-424-5p in the cytoplasm. To determine and confirm the target sites of lncRNA-miRNA pairs and miRNA-mRNA pairs, we used miRanda [19] (Figure 1F). As predicted by lncLocator2 [20], the lncRNAs *DUAXP8*, *CDKN2B-AS1*, *lnc-RSG5-1*, and *MCM2AP-AS1* were primarily found in the cytoplasm (Figure 1G). The beneficial correlations were also demonstrated (Figure 1H and Supplementary Figure 1B–1F).

As a result, we chose two hub regulatory networks from the refined network, particularly the network that is *CBX2*/*CEP5*/*MCM2*-centered.

### Construction and validation of the *CBX2/CEP55*-featured prognostic model

**Figure 2:**
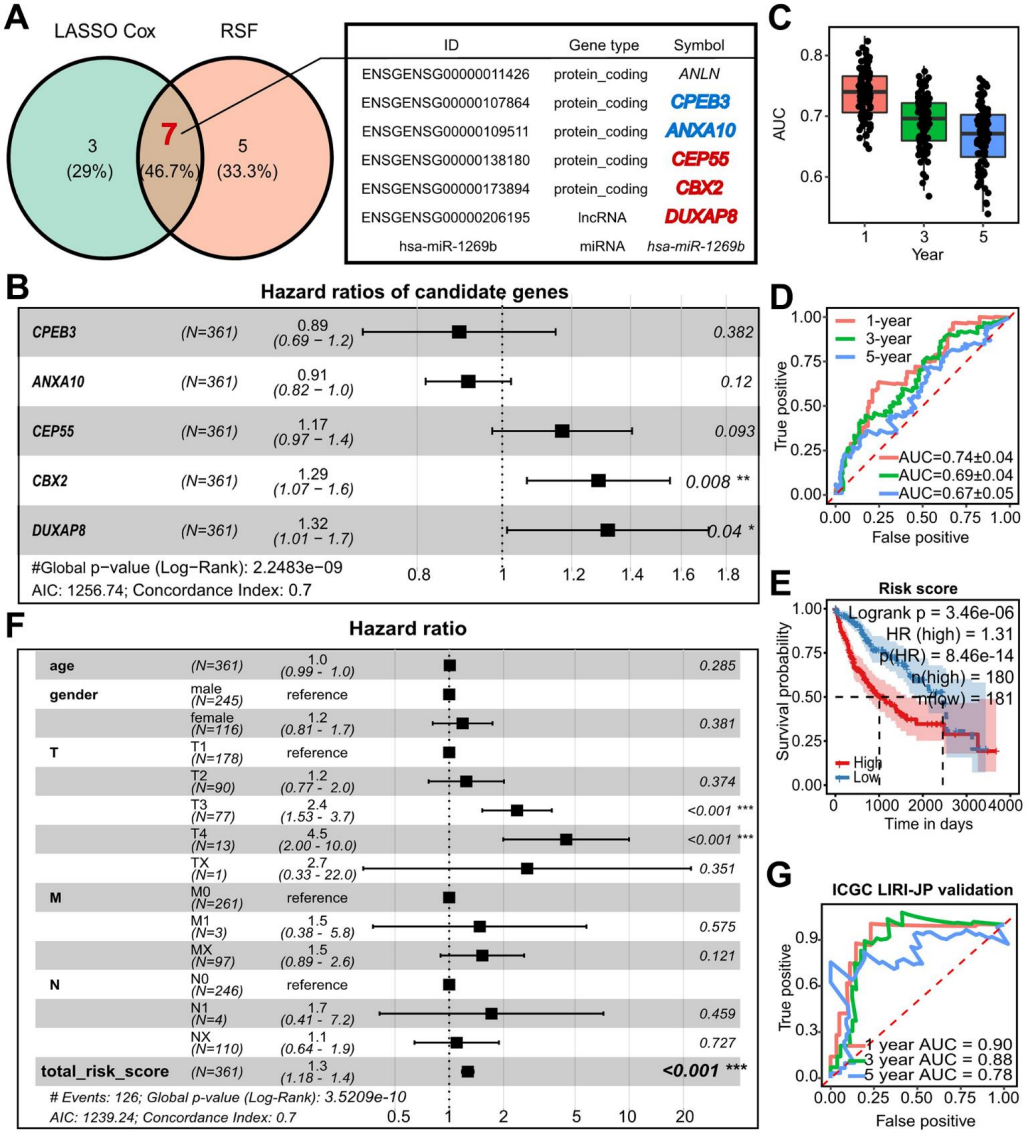
**Establishment and refinement of *DUXAP8*/*CBX2*/*CEP55*-centered prognostic model.** (**A**) Shared model candidate survival-related RNAs from which was selected with Lasso Cox and RSF from the refined regulatory networks. (**B**) Forest plots of multivariate Cox showed the hazard ratio (HR), 95% confidence interval (CI), and corresponding P-values of model-used *CPEB3*, *ANXA10*, *CEP55*, *CBX2*, and *DUXAP8* (**C**) Time-dependent AUC within 5 years of a prognostic model related to 5-survival genes using two-fold cross validation with 50 randomly repeated replications. (**D**) The examples of time-dependent ROC at 1-, 3-, and 5-year corresponding to the AUC in (**C**). (**E**) Kaplan-Meier plots of the risk score predicted with the prognostic model in TCGA-LIHC. The high and low risk group was determined with the median of risk score. P value was calculated by log-rank test. (**F**) Forest plots of multivariate Cox showed HR and p value of TNM, age, gender and risk score. (**G**) Time dependent ROC and AUC at 1-, 3-, and 5-year predicted with external validation using ICGC-LIRI-JP by 5-survival-gene prognostic model. RSF, Random Survival Forest; AUC, area under the curve; ROC, receiver operating characteristic curve; HR, hazard ratio.

We first looked at the effect on survival time to investigate the clinical relevance of the RNAs in the refined network. We used univariate Cox analysis and found that 0/8 miRNA, 6/25 lncRNA, and 20/42 mRNA all had an impact on the survival time (Supplementary Figure 3 and Supplementary Table 2). To further narrow down the list of essential RNAs, we used Lasso Cox and Random Survival Forest (RSF). This led to the overlap of 7 RNAs. *ANLN*, on the other hand, failed the Cox-PH test, and has-miR-1269b significantly failed the univariate Cox analysis. As a result, we identified 5 key prognostic RNAs, including *CPEB3*, *ANXA10*, *CEP55*, *CBX2*, and *DUXAP8* (Figure 2A and Supplementary Figure 4A). *DUXAP8* and *CBX2* were found to be independent prognostic genes by multivariate Cox analysis, and a prognostic model with a C-index of 0.7 was created (Figure 2B). The established prognostic model and nomogram demonstrated observable performance as indicated by calibration plots and time-dependent AUC greater than 0.7 (Supplementary Figure 4B–4D). We used two techniques to test the prognostic model’s robustness: (1) two-fold cross-validation performed 50 times at random, and (2) external validation using the ICGC-LIRI-JP dataset. Time-dependent AUC values greater than 0.7 and 0.8, respectively, in two-fold cross-validation of LIHC and LIRI-JP external validation suggested the prognostic model with 5 genes performed well. (Figure 2C–2E). A worse prognosis was independently linked to the risk score determined by the prognostic model (Figure 2F, 2G).

Surprisingly, we noticed that *DUXAP8*, *CBX2*, and *CEP55* appeared in both the prognostic model and the hub of the regulatory network. Additionally, a worse prognosis was indicated in LIRI-JP by higher expression of *CBX2* and *CEP55* (Supplementary Figure 4E, 4F). The worst overall survival was seen in patients with double higher expression of *CBX2* and *CEP55* (Supplementary Figure 4G). Notably, there was a significant correlation between the biomarker for HCC *AFP*, *CBX2*, and *CEP55* (Supplementary Figure 12G). Along with an increase in *CBX2* and *CEP55*, the stepwise stage and tumor grade also increased (Supplementary Figure 12G). As a result, for the step analysis that follows, *CBX2* and *CEP55* were picked as leading actors.

### Abnormal *CBX2* and *CEP55* expression in HCC were validated in numerous studies

We gathered numerous cohorts and carried out a differential expression analysis in order to confirm the abnormal expression of *CBX2* and *CEP55*. In the following GSEs: GSE138485, GSE144269, GSE148355, GSE54236, GSE22508, GSE25097, GSE63898, GSE112790, and ICGC LIRI-JP, higher expression of *CBX2* in HCC compared to adjacent tissue has been approved. These cohorts showed a *CEP55* trend that was comparable to the *CBX2* trend (Supplementary Figure 6A–6G). The expression profile of the mouse liver tumor model from GSE116463 also revealed this outcome (Supplementary Figure 6P). Immunohistochemistry staining (IHC) from the HPA database also showed similar deregulation of *CBX2* and *CEP55* expression (Supplementary Figure 5K). Intriguingly, we found that *CBX2* and *CEP5*5 expressed more in para-cancerous tissue in GSE25097 compared to healthy tissue, suggesting that abnormally expressed *CBX2* and *CEP55* may be responsible for the development of cancer (Supplementary Figure 6D).

### Genomic and epigenetic alternation regulated aberrant *CBX2*/*CEP55* expression

**Figure 3:**
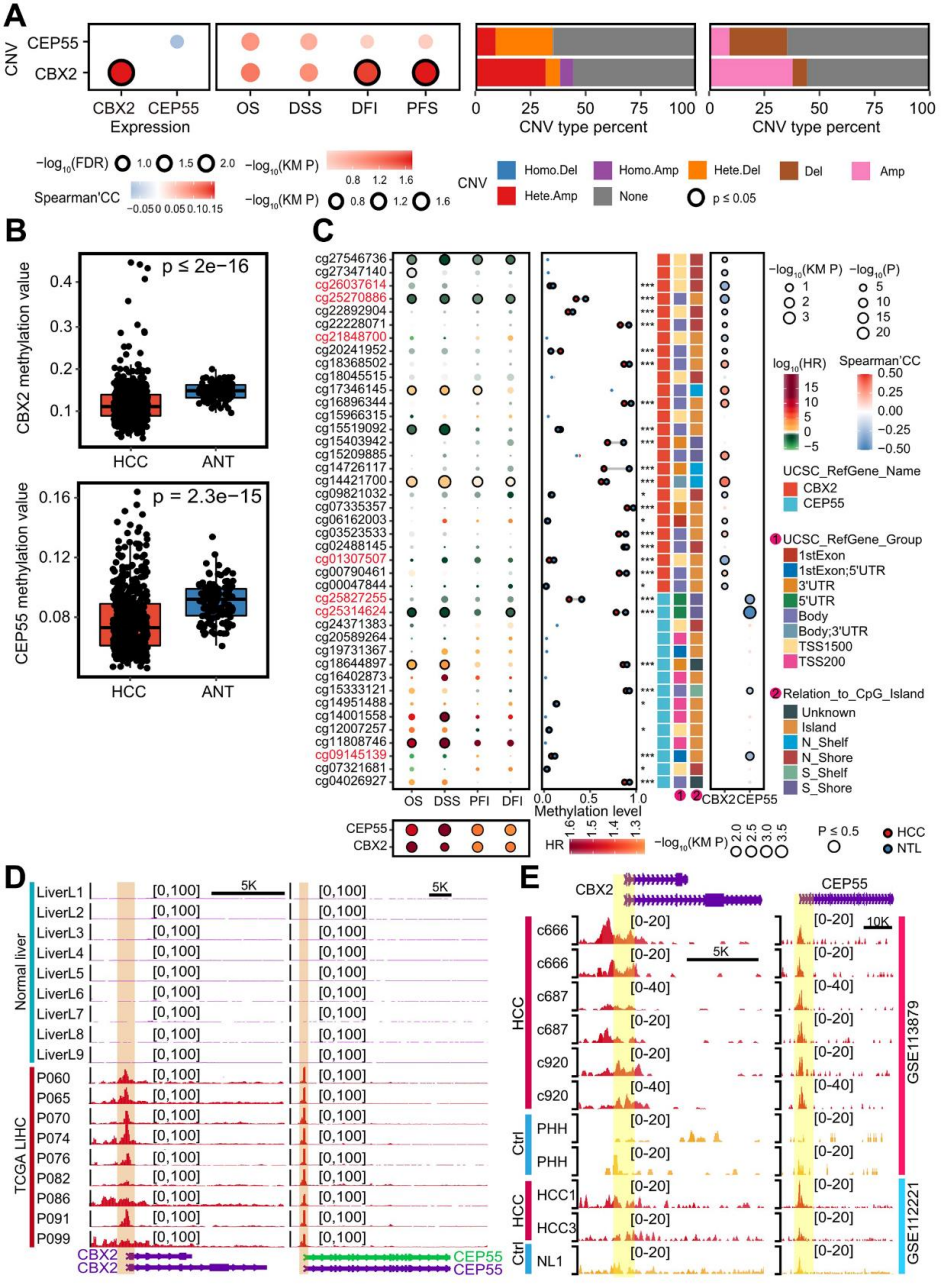
**Genomic and epigenomic alternations enhanced *CBX2* and *CEP55* expressions.** (**A**) From left to right, the figures were the Spearman’s correlation of CNV and the corresponding expression, the impact of CNV on patients’ survival time, and the percent of *CBX2* and *CEP55* CNV type detailly and broadly. KM P indicated the P-value computed with log-rank test. (**B**) Methylation level of *CBX2* and *CEP55* in HCC and adjacent tissues. P-value was performed using Wilcox rank sum test. (**C**) From left to right, the figures were the effects of methylation sites on *CBX2* and *CEP55* on patients’ survival, the mean methylation level of *CBX2* and *CEP55* in HCC and adjacent tissues, and the Spearman’s correlation between methylation level of methylation sites and expression level. The P-value reflecting differential methylation sites was derived from the Wilcox rank sum test. KM P indicated the P-value computed with log-rank test and the median of methylation level or expression level was utilized to classify the high and low group. (**D**) Chromatin accessibility signals on *CBX2* and *CEP55* in normal livers and HCC. (**E**) H3K4me3 signals on *CBX2* and *CEP55* in normal livers and HCC. HR, hazard ratio. *, P ≤ 0.05; **, P ≤ 0.01; ***, P ≤ 0.001; OS, Overall Survival; DSS, Disease Specific Survival; DFI, Disease Free Interval; PFI, Progression Free Interval.

We first characterized the genomic alternations in order to investigate the possible causes of the abnormal expression of *CBX2* and *CEP55*. For *CBX2* and *CEP55*, no more than five single nucleotide mutations were found (Supplementary Table 3). In terms of copy number variation (CNV), *CBX2* underwent major amplification, whereas *CEP55* underwent major deletion. However, only CNV of *CBX2* had a favorable impact on self-expression (Figure 3A and Supplementary Figure 5A). Additionally, we found that the CNV of CBX2 had a significant impact on both the Disease Free Interval (DFI) and Progression Free Survival (PFS) (Figure 3A and Supplementary Table 4).

One method for controlling gene expression was thought to be DNA methylation. Additionally, we discovered that the levels of three DNA methyltransferases (*DNMT1*, *DNMT3A*, and *DNMT3B*) were significantly higher in the *CBX2*^high^ tumor than the *CBX2*^low^ tumor and the *CEP55*^high^ tumor than the *CEP55*^low^ tumor (Supplementary Figure 5B–5G). Additionally, we found that adjacent tissue had significantly higher levels of methylation for *CBX2* and *CEP55*, indicating coincidentally lower expression. Cohorts GSE113019, GSE44909, GSE44909, and GSE37988 also supported this outcome (Figure 3B and Supplementary Figure 6H–6O). Next, we determined the Spearman Correlation coefficient and patient survival time using Univariate Cox analysis to characterize the effect of particular methylation sites on gene expression. Differential methylation sites at cg26037614, cg22892904, cg09821032, and cg01307507 revealed hyper-methylation harboring promoter-associated (TSS1500) CpG island, displaying a strong inverse relationship with *CBX2* expression and a favorable prognosis for patient survival (Figure 3C). We also discovered three hyper-methylation CpG islands at the promoter-associated (5’UTR) CpG island for *CEP55*, cg25827255, cg25314624, and cg09145139, which have an adverse effect on the expression of *CEP55* and increase the survival time of patients (Figure 3C). The MEXPRESS database was used to verify this finding [21] (Supplementary Figure 6Q).

The accessible chromatin throughout the genome demonstrated the co-operative regulation of gene expression by enhancers, promoters, and chromatin-binding factors [22]. Histone H3 lysine 4 was tri-methylated (H3K4me3), designating promoters associated with transcription start sites, while H3K4 was mono-methylated (H3K4me1) and histone H3 lysine 27 was acetylated (H3K27ac), designating enhancers to activate gene expression [23, 24]. We looked at the H3K27ac signal, H3K4me3, H3K4me1, and chromatin accessibility of *CBX2* and *CEP55*. We discovered a higher chromatin accessibility signal in HCC tissue and HCC cell lines compared to normal liver tissue on the *CBX2* and *CEP55* loci (Figure 3D and Supplementary Figure 5H). Likewise, similar patterns were seen for H3K4me3, H3K4me1, and H3K27ac (Figure 3E and Supplementary Figure 5H–5J). Thus, in addition to miRNA, genomic and epigenomic changes may also control the aberrant expression of *CBX2* and *CEP55*.

### *CBX2*/*CEP55* affected the cell cycle process

**Figure 4:**
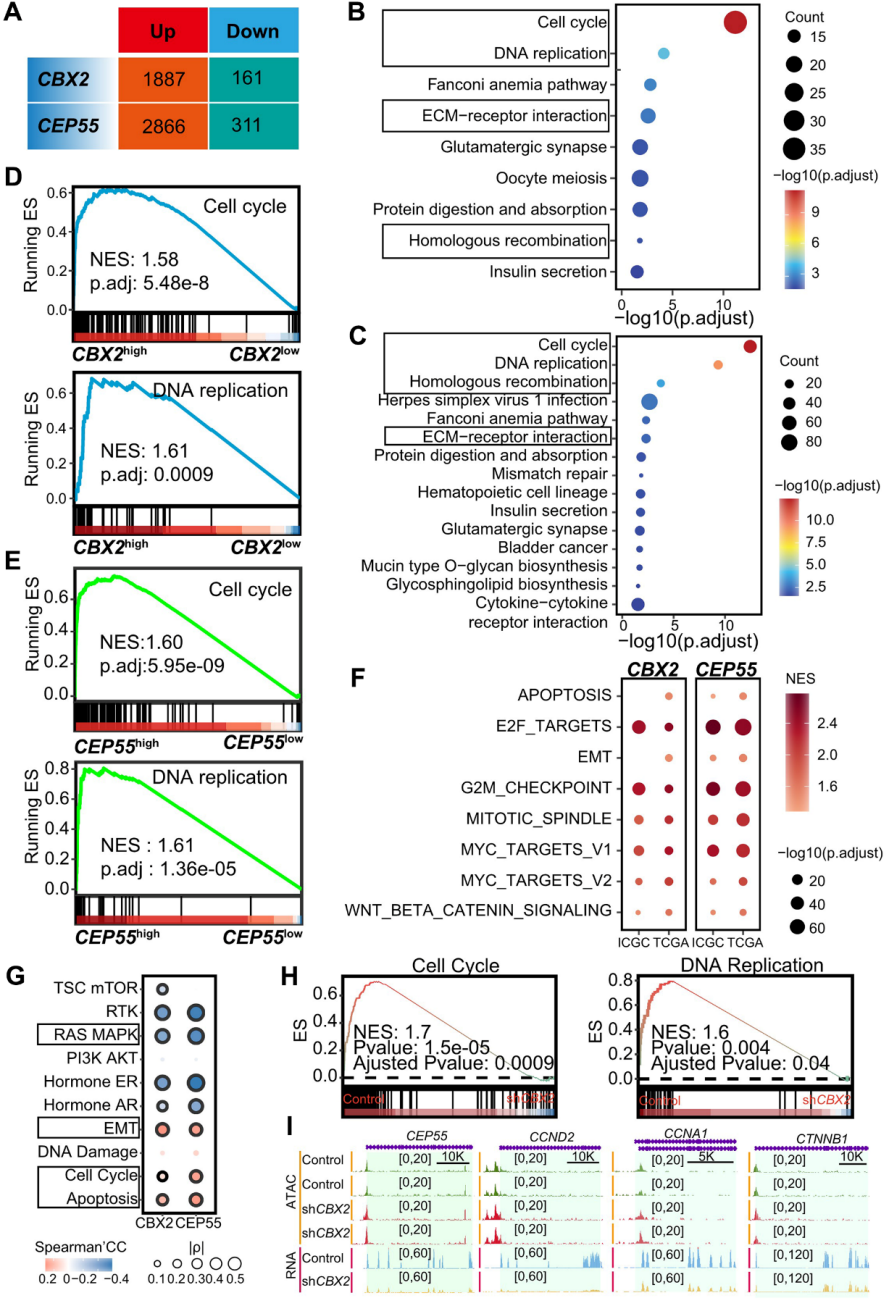
***CBX2* and *CEP55* affected the cell cycle.** (**A**) Summary of differential expressed genes identified using DESeq2 in *CBX2*-stratified and *CEP55*-stratified tumors. The genes in Up indicated the higher expression in *CBX2*^high^ or *CEP55*^high^ tumors whereas conversely for those in Down. (**B**, **C**) Enriched KEGG in 1887 *CBX2*-related up-regulated genes (**B**) and 2866 *CEP55*-related up-regulated genes (**C**) in stratified tumors. (**D**, **E**) GSEA analysis of *CBX2*-related (**D**) and *CEP55*-related (**E**) KEGG pathway. (**F**) GSEA analysis of *CBX2*-related and *CEP55*-related cancer hallmarks. (**G**) The Spearman’s correlation between pathway activity score and *CBX2*/*CEP55* expression. (**H**) GSEA analysis of *CBX2* knockdown -related KEGG pathways. (**I**) RNA and ATAC tracks of cell cycle-related genes in sh*CBX2* and WT group.

To determine the potential mechanism of *CBX2* and *CEP55* contributing to tumorigenesis, we stratified with the median expression of *CBX2* and *CEP55* and identified 1338 *CBX2*-related and 3177 *CEP55*-related differentially expressed genes, respectively (Figure 4A). Up-regulated genes in *CBX2*^high^ tumors were enriched for cell cycle-related terms like nuclear division and mitotic nuclear division as well as extracellular matrix organization (Supplementary Figure 7A). GSEA for the GO gene set showed comparable results (Supplementary Figure 7B). The expression of genes involved in the cell cycle, DNA replication, ECM receptor interaction, and homologous recombination pathways was increased (Figure 4B). This outcome was seen when using the KEGG gene set in GSEA analysis (Figure 4D). Drug metabolism and fatty acid metabolism were enriched for genes up-regulated in *CBX2*^low^ tumors (Supplementary Figure 7C, 7D). In tumors that were *CEP55*-stratified, a similar outcome was seen (Figure 4C, 4D and Supplementary Figure 8A–8D). The cell cycle and DNA replication were strongly correlated with *CEP55* expression (Figure 4C, 4D). Along with being positively correlated with cell cycle-related pathways (such as E2F targets, G2/M checkpoint, mitotic spindle, MYC targets V1 and V2) and epithelium mesenchymal transition, DEGs related to *CBX2* and *CEP55* were also found in signature gene sets (Supplementary Figure 9A).

To validate that *CBX2* and *CEP55* expression could regulate the cell cycle pathway, we first calculated the Pearson Correlation Coefficients (PCC) between all genes and *CBX2* or *CEP55* in LIHC and LIRI-JP cohorts. We also identified the cell cycle pathway, DNA replication pathway, ribosome pathway, cell cycle-related hallmark (E2F targets, G2/M checkpoint, mitotic spindle, MYC targets V1, MYC targets V2), EMT, apoptosis, and WNT beta-catenin signaling using GSEA with the KEGG and hallmark gene set based on the PCC (Figure 4F and Supplementary Figure 9B–9E). The majority of the genes closely linked to the expression of *CBX2* and *CEP55*, including those in the CENP and KIF families, were found to be related to the cell cycle (Supplementary Figure 9H, 9I). And many of these genes—including *TOP2A*, *ORC6*, *MCM2*, and *PLK1* - were crucial as determined by Chronos scores (Supplementary Figure 10A, 10B). Furthermore, we observed that *CEP55* displayed a high PCC with *MKI67*, which was regarded as a proliferation marker (Supplementary Figure 9I).

To further certify *CBX2* expression regulated the cell cycle pathway, we reanalyzed *CBX2*-KO datasets including GSE193477 (human AML U937), GSE112227 (mouse long bone), and GSE156413 (mouse embryonic fibroblast). We discovered the cell cycle pathway and cell cycle-related pathways (DNA replication, *E2F* targets, G2/M checkpoint) were enriched in the *CBX2* WT group (Figure 4H and Supplementary Figure 9F, 9G). For instance, in GSE156413, *CBX2* KO groups reduced *Ccna1* chromatin accessibility and expression level in comparison to WT (Supplementary Figure 9G). In GSE193477, we found that *CBX2* KO reduced the expression of cell cycle-related genes like *CCND2* and *CCNA1*, but had few effects on chromatin accessibility (Figure 4I).

The Spearman Correlation Coefficient (SCC) between the two genes and the cancer-related pathway score revealed that *CBX2* and *CEP55* expression positively regulate the cell cycle, apoptosis, and EMT while negatively modulating the RAS MARK pathway (Figure 4G). Through the MAPK signaling pathway, *CBX2* has been shown to influence chromatin accessibility and promote AML [25]. EMT may be brought on by the death of cancer cells [26].

The overall result showed that *CBX2* and *CEP55* may regulate the cell cycle process to support HCC.

### CBX2 impacted the cell cycle directly or indirectly

**Figure 5:**
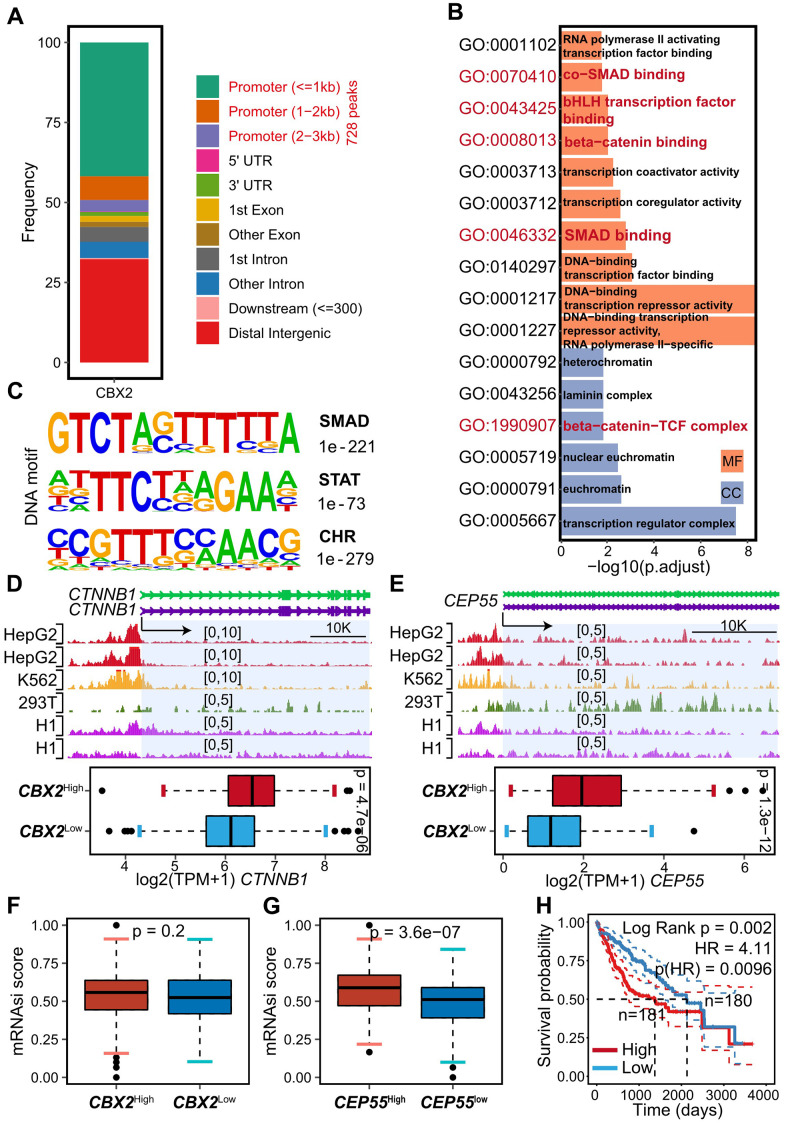
**CBX2 regulated *CEP55* and *CTNNB1* directly.** (**A**) Summary of genomic distribution of CBX2 peaks. (**B**) Enriched GO terms in genes associated with gene promoter CBX2 peaks. (**C**) DNA motifs enriched within genes associated with gene promoter CBX2 peaks determined by HOMER motif analysis. (**D**, **E**) Tracks of CBX2 peaks on *CEP55* (D) loci and *CTNNB1* (E) loci and the corresponding expression between *CBX2*-statified tumors. Statistical significance was calculated using the two-sided Wilcoxon test. (**F**, **G**) mRNAsi distribution between *CBX2*-stratified (**F**) and *CEP55*-stratified (**G**) tumors. Statistical significance was calculated using the two-sided Wilcoxon test. (**H**) Kaplan-Meier plots of mRNAsi. The high and low group was classified with the median of risk score. P-value was computed with log-rank test.

It was intriguing to see that *CBX2* knockout reduced *CEP55* expression (Figure 4I), which inspired us to research the potential functions of *CBX2*. The CBX2 ChIP-seq of HepG2 was therefore reanalyzed, and we obtained IDR-confirmed 1305 peaks, of which 728 peaks were found at the gene promoter (Figure 5A). Surprisingly, cell cycle-associated ontologies with enriched content included co-SMAD binding, bHLH transcription factor binding, beta-catenin binding, SMAD binding, and beta-catenin-TCF complex (Figure 5B), indicating that CBX2 might indirectly control the cell cycle process by interacting with other transcription factors. The discovery of the SMAD and STAT motifs (Figure 5C) suggests that CBX2 may work with the STAT and SMAD TF family to control the cell cycle. The CHR (cell cycle genes homology region) motif (Figure 5C), on the other hand, was thought to be a suppressor of the cell cycle and was a binding site recognized by the DREAM complex, indicating CBX2 could regulate the cell cycle by repressing or competing with the DREAM complex [27–29].

CBX2 consistently bound to the *CEP55* promoter as shown by CBX2 ChIP in the HepG2, 293T, K562 and H1 cell lines, and ectopic expression of *CBX2* in HCC increased the transcription of *CEP55* (Figure 5E). The plasma membrane and nucleoplasm, respectively, were the locations of *CBX2* and *CEP55*, according to multiplexed immunofluorescence images downloaded from the HPA (Supplementary Figure 11A, 11B). Additionally, we observed that CBX2 bound to the *CTNNB1* promoter and increased the expression of *CTNNB1*, which encodes beta-catenin (Figures 4I, 5D), in accordance with GO:0008013 enrichment (Figure 5B) and GSEA enrichment results (Figure 4F). Beta-catenin had been confirmed to aggravate hepatocarcinogenesis and promote cancer stem cell properties [30], which enforced us to explore the relationship between *CBX2*, *CEP55* and cancer stem cells. A higher mRNAsi score was noted in *CBX2*-high tumors and *CEP55*-high tumors when we calculated the mRNAsi score to assess the stem index of tumors with the *CBX2*-stratified and *CEP55*-stratified expression profiles (Figure 5F, 5G). Besides that, the prognosis of the patients with higher mRNAsi was worse (Figure 5H). According to this research, *CBX2* could control *CEP55* or *CTNNB1* to control the characteristics of cancer stem cells that are consistent with more aggressive migration.

### *CBX2* drove a CSC-like phenotype

**Figure 6:**
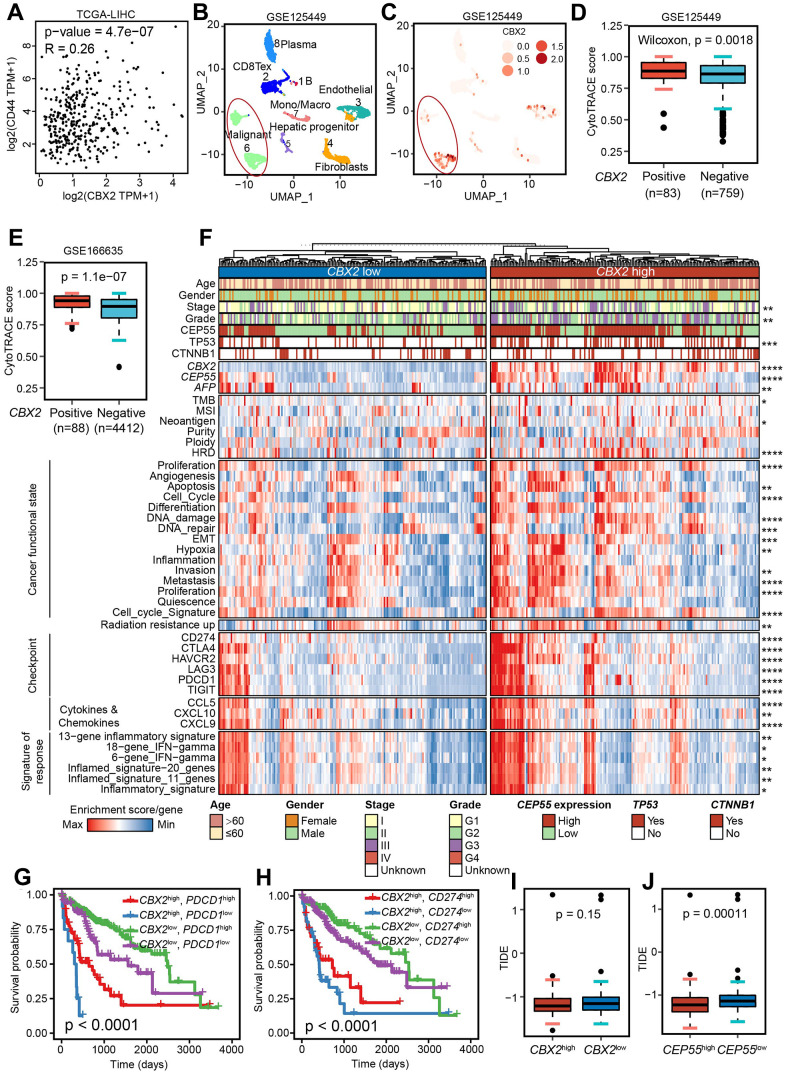
***CBX2* shaped diverse functional states and enhanced immunotherapy response.** (**A**) The Pearson correlation between *CBX2* and *CD44* in TCGA-LICH dataset. (**B**, **C**) UMAP showing the cell clusters (**B**) and distribution of *CBX2* (**C**) in GSE125449. (**D**, **E**) Distribution of CytoTRACE score between CBX2-positive and –negative malignant cells from GSE125449 (**D**) and GSE166635 (**E**). P-value was calculated using the two-sided Wilcoxon test. (**F**) Heatmap representation of the main functional states, immunotherapy response predictors, representative molecular and immune characteristics in *CBX2*^high^ tumors and *CBX2*^low^ tumors. (**G**, **H**) Kaplan-Meier plots of *CBX2* combined with *PDCD1* (**G**) and *CD274* (**H**). P-value was computed with log-rank test. (**I**, **J**) Distribution of predicted TIDE score between *CBX2*-stratified (**I**) and *CEP55*-stratified (**J**) tumors. P-value was calculated using the two-sided Wilcoxon test.

To further validate whether *CBX2* and *CEP55* affect malignant cell stemness, we reanalyzed the public datasets related to HCC CSC and observed that *CBX2* and *CEP55* levels were higher in cancer stem cells that were CD133, ALDH, or CD44-labeled (Supplementary Figure 11C). Additionally, both CBX2 and CEP55 demonstrated a high Pearson correlation with a CSC marker *CD44* (Figure 6A and Supplementary Figure 12D). Three scRNA-seq datasets GSE125449, GSE140228 and GSE166635 downloaded from the TISCH2 database [31] were analyzed, and we found that *CBX2* was primarily expressed in cancerous tumor cells while *CEP55* was primarily expressed in T cells that were proliferating (T prolif), whose marker gene *MKI67* (Figure 6B, 6C and Supplementary Figure 12A, 12B). Additionally, it was also discovered that CEP55 is expressed in regulatory T-cells (Tregs) and tumor cells (F). The scRNA-seq data from healthy liver tissue downloaded from the HPA served as validation for this finding that *CBX2* in hepatocytes and endothelial cells whereas *CEP55* in T cell. (Supplementary Figure 12C). CytoTRACE was utilized to infer intercellular activity and determine the differentiation status of cells with a matrix that counts single cells. A higher CytoTRACE score indicate a higher level of cell stemness. A total of 842 malignant cells from GSE125449 and 4500 malignant cells from GSE166635 were used to validate the influence of CBX2 on tumor stemness using CytoTRACE. The findings showed that CBX2-positive malignant tumors demonstrated significantly elevated levels of stemness rather than CEP55-positive (Figure 6D, 6E and Supplementary Figure 12F). Moreover, we conducted differential gene identification and GSEA analysis on CBX2-positive and -negative malignant cells derived from GSE125449. The results revealed significant enrichment of glycolysis, hypoxia, EMT, cell-cycle-related MYC target V1, E2F target, and G2M checkpoint in *CBX2*-positive malignant cells (Supplementary Figure 12E). These findings suggested that the expression of *CBX2* amplifies tumor malignancy and facilitates cancer progression.

Generally, CSC was characterized by the unlimited proliferation, high drug resistance, promotion of heterogeneity formation, and metastatic recurrence. Different tumor phenotypes, reflecting cancer-related functional states, were produced throughout the entire tumor evolution as a result of functionally heterogeneous cancer cells cooperating or competing [12]. We then investigate *CBX2* and *CEP55*’s effects on cancer-related functional states. Apoptosis, EMT, and cell cycle scores were all noticeably higher in *CBX2*^high^ and *CEP55*^high^ tumors (Figure 6F and Supplementary Figure 12G). In addition, *CBX2* and *CEP55* might promote the growth, invasion, wound healing score, differentiation, and metastasis of tumors (Figure 6F and Supplementary Figure 12G). Taken together, *CBX2* drove cancer stem cell properties.

### *CBX2*/*CEP55*, affected immune infiltration, enhanced radio-resistance and predicted immunotherapy response

A highly structured ecosystem of TME can shape cancer cells’ capacity for proliferating, migrating, developing drug resistance, or responding to immunotherapy. Next, we evaluated the stromal score, immune score, and TME score using the ESTIMATE algorithm, and found that *CBX2* had no impact on any of them while *CEP55* only improved immune infiltration (Supplementary Figure 14A, 14B). The signature score from Thorsson et al. [13] supported the observations (Supplementary Figure 14A, 14B). More specifically, we estimated the cell fraction using CIBERSORT, EPIC, QUANTISEQ and MCPCOUNTER methods and assessed the effect of *CBX2* and *CEP55* on the cells by computing the Spearman Correlation Coefficient. We discovered that cancer-associated fibroblast (EPIC, MCPCOUNTER) strongly connected with *CBX2* and *CEP55*, consistently with ECM enrichment (Supplementary Figure 13G, 13I and Figure 4B, 4C). Additionally, tumors with high levels of *CBX2* and *CEP55* have higher levels of immunosuppressive cells such as regulatory T cells and myeloid-derived suppressor cells (Supplementary Figure 13E, 13F, 13H, 13J).

In order to understand the regulation of *CBX2* or *CEP55* on TME, we looked at the distributions of MHC-related genes, immune stimulators, and immune inhibitors in LIHC. *CBX2*^high^ and *CEP55*^high^ tumors were found to have higher levels of the majority of MHC, immune-stimulating, and immune-inhibiting genes, including immune inhibitor *PDCD1*, *CD274*, and immune stimulator *TNFRSF9* (Supplementary Figure 14A, 14B). This suggests that these tumors had complex TME. Additionally, *CBX2* and *CEP55* had a significantly greater impact on tumor patient survival than *PDCD1* and *CD274* (Figure 6G, 6H).

Neoantigen, microsatellite instability (MSI), and tumor mutation burden (TMB) were considered new indicators of immunotherapy efficacy [13]. Since TMB and neoantigen levels were higher in *CBX2* while MSI levels were higher in *CEP55*, two molecules could improve immunotherapy (Figure 6F and Supplementary Figure 12G). In order to further validate it, we computed the ssGSEA score using the inflammatory signature, the inflammatory signature, and the IFN-gamma signature, all of which have been shown to accurately predict the outcome of immunotherapy [15, 16]. *CBX2*^high^ and *CEP55*^high^ tumors with high signature scores had better outcomes from immunotherapy (Figure 6F and Supplementary Figure 12G). The Tumor Immune Dysfunction and Exclusion (TIDE) score was used to verify the findings; a higher TIDE score suggested a higher probability of cancers evading the immune system [32]. For tumors with high levels of *CBX2* and *CEP55*, we found lower TIDE scores, indicating a better response to immunotherapy (Figure 6I–6J and Supplementary Figure 13A–13D). Additionally, it was discovered that tumors with elevated levels of *CBX2* and *CEP55* had higher concentrations of the chemokines *CCL5*, *CXCL9*, and *CXCL10* linked to immunotherapy response [33] (Figure 6F). All of the results demonstrated *CBX2*^high^ and *CEP5*^high^ tumors could benefit from immunotherapy.

The effect of *CBX2* and *CEP55* on radiation resistance was then investigated. Higher radiation resistance up-regulated gene enrichment was seen in *CBX2*^high^ and *CEP55*^high^ tumors (Figure 6F and Supplementary Figure 12G). Additionally, based on the DepMap expression profiles, we used oncoPredict to forecast the sensitivity of drugs for *CBX2*-stratified tumors. Patients with high levels of *CBX2* were compatible with drugs like BAY 87-2243 (a HIF-1 inhibitor), Binimetinib (a MEK inhibitor), Voreloxin (a topoisomerase II inhibitor), Floxrudidine (oncology antimetabolites), Lenvatinib, Regorafenib, Paclitaxel, and Indisulam but not Sorafenib, demonstrating *CBX2* affects the effectiveness of medications (Supplementary Figure 10C).

Therefore, overexpressing *CBX2* and *CEP55* may cause cancer cells to develop cancer stem cell-like phenotypes with high levels of invasion, metastasis, and radiation resistance. However, immunotherapy may be advantageous for it.

### *CBX2*/*CEP55*, as drug targets in pan-cancer

**Figure 7:**
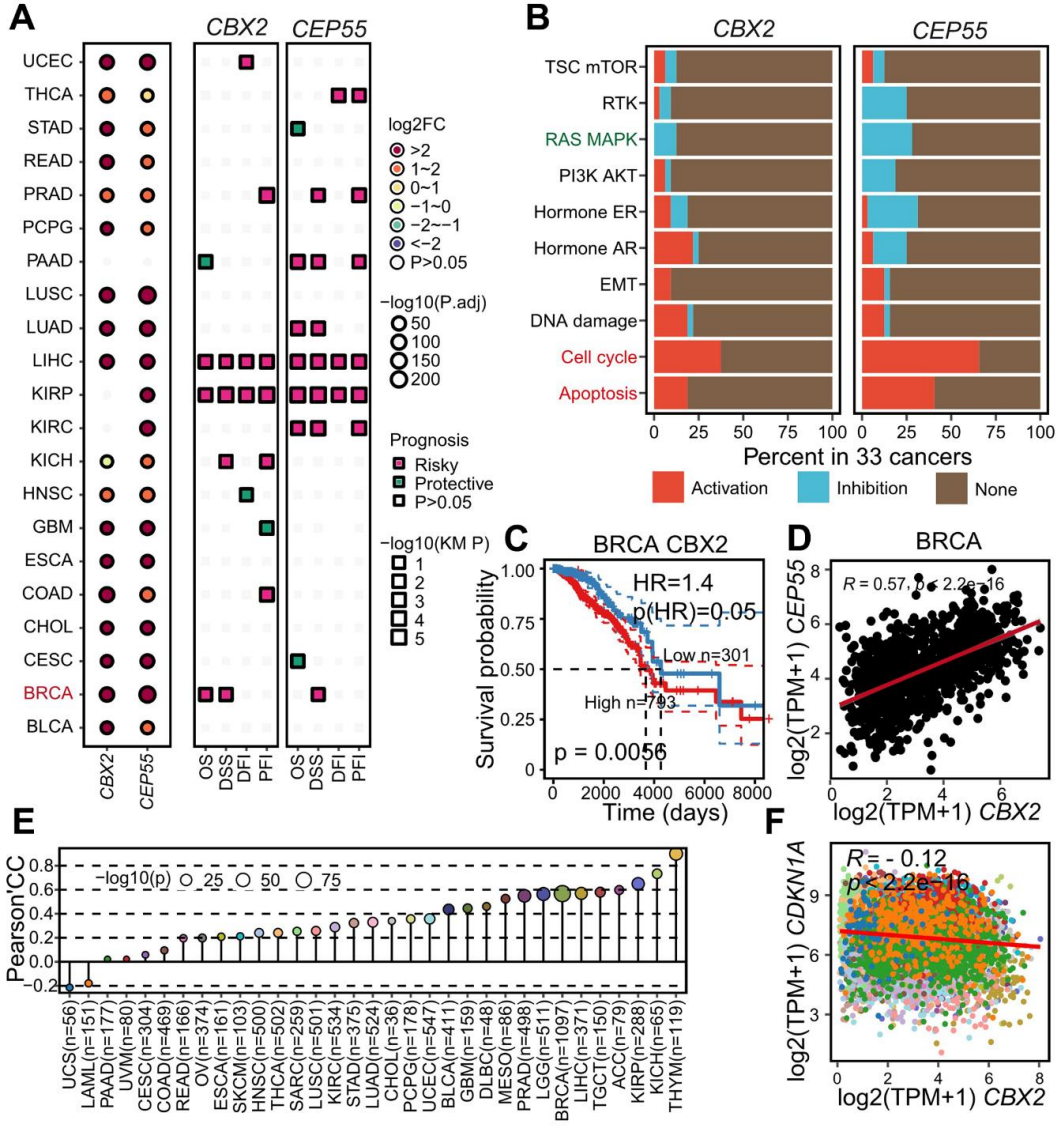
**Aberrantly expressed *CBX2* and *CEP55* as drug targets as pan-cancer level.** (**A**) Summary of *CBX2* and *CEP55* expression pattern differences and their impact on tumor patient survival time (OS, DSS, DFI, PFI) across 21 cancers. Prognosis was inferred with hazard ratio, “risky” indicated HR > 1 whereas “protective” suggested HR < 1 (**B**) Summary of pathway activation or inhibition by *CBX2* and *CEP55* across 33 cancers. “Activation” represented significantly positive Spearman’s correlation conversely “Inhibition” indicated the significantly negative. (**C**) Kaplan-Meier plots of *CBX2* expression in BRCA. High and Low groups were determined by the *CBX2* expression cutoff computed by surv_cutpoint function in survminer package. P-value was computed with log-rank test. (**D**) Pearson’s correlation between the expression of *CBX2* and *CEP55* in BRCA, an example of pan-cancer. (**E**) Pearson’s correlation between the expression of *CBX2* and *CDKN1A* with all tumors. OS, Overall Survival; DSS, Disease Specific Survival; DFI, Disease Free Interval; PFI, Progression Free Interval. (**F**) Pearson’s correlation of *CBX2* and *CDKN1A* expression level in pan-cancer.

Surprisingly, we performed the differential gene analysis in another 20 cancers and found *CBX2* and *CEP55* up-regulated in 16 and 18 of 20 cancers. Additionally, BRCA and KIRP patients with high-expressed *CBX2* showed worse overall survival while BRCA, KIRP, KIRC, LUAD and PAAD patients with high-expressed *CEP55* showed worse survival (Figure 7A, 7C). We also observed that *CBX2* and *CEP55* enhanced the cell cycle and apoptosis pathway and inhibited the RAS MAPK pathway. Next, we computed the PCC between *CBX2* and *CEP55* to validate their positive regulatory relationship across cancers and confirmed that a significantly positive correlation greater than 0.2 was observed in 27/33 cancers, especially in THYM (Figure 7E, 7D).

*CBX2* and *CEP55* had been demonstrated to promote HCC through the cell cycle route. A greater *TP53* mutation rate was seen in *CBX2*^high^ tumors, indicating a potential P53 function loss (Figure 6F and Supplementary Figure 12G). Additionally, we pointed out that the deletion of *CBX2* could lower the expression of the genes *CCND2*, which codes for cycling D2, and *CCNA1*, which codes for cycling A1, both were the crucial regulators of the cell cycle (Figure 4I). The tumor suppressor gene *CDKN1A*, which codes for the cell cycle regulator p21, could prevent CDK from kinase activity. At the level of all cancers, we noticed that *CBX2* and *CDKN1A* exhibited a negative trend, strengthening CBX2’s ability to regulate the cell cycle (Figure 7F).

## DISCUSSION AND CONCLUSION

Due to its poor prognosis and high aggressiveness, HCC is one of the main causes of cancer mortality in the world [2]. Although patients with HCC have received a variety of therapeutic approaches, the prognosis is still dismal [3, 34]. Finding new therapeutic targets and improving patient outcomes for this condition requires a clear understanding of the molecular mechanisms and processes underlying the pathogenesis of HCC. It had been suggested that the regulatory networks were involved in the development and progression of HCC [6].

Using the fine-tuned triple regulatory networks and reliable prognostic model, we thoroughly identified the key up-regulated genes *CBX2* and *CEP55* in this study. Numerous cohorts showed abnormal *CBX2* and *CEP55* expression, demonstrating the persistence and significance of HCC tumorigenesis and progression. We verified the cause of *CBX2* and *CEP55*’s aberrant expression. Amplification of CNV, DNA hypo-methylation, open chromatin accessibility, and high active marks signals (H3K4me3, H3K4me1, and H3K27ac), in addition to miRNA miR-424-5p (Figure 1E), cause the expression of *CBX2* and *CEP55* to be higher in tumors than in adjacent tissues. Additionally, by specifically targeting *E2F7*, the tumor suppressor miR-424-5p regulated the cell cycle and decreased proliferation [35]. However, it was *CBX2* or *CEP55* and miR-424-5p that were first linked. Meanwhile, it was discovered that function as ceRNA to regulate *CBX2* and *CEP55* first included *DUXAP8*, *MCM3AP-AS1*, and *CDKN2B-AS1*.

We identified differentially expressed genes and carried out enrichment analysis to investigate the potential mechanism that may be promoting the HCC of *CBX2* and *CEP55*. Apoptosis and the cell cycle pathway had a strong positive correlation with *CBX2* and *CEP55*. The results were supported by *CBX2* knockout in numerous cohorts. Nevertheless, *CBX2* knockout reduced the expression and only slightly the chromatin accessibility of the genes involved in the cell cycle (Figure 4I).

The cell cycle was kept in working order by *CEP55* (Centrosomal protein 55) and *CBX2* (Chromobox homolog 2, also termed cell division cycle associated 6). We used ChIP-seq in HepG2 to investigate how CBX2 improved the cell cycle. Surprisingly, we discovered that CBX2 could bind to the promoter of *CEP55* and *CTNNB1*, increasing the expression of both genes (Figures 4I, 5D, 5E). Additionally, CBX2 could work in conjunction with the SMAD transcription factor family and beta-catenin to encourage gene expression. However, we also discovered the CHR motif, which the DREAM complex recognized and bound to stop the cell cycle [27, 28]. In most cases, PRC1’s core subunit CBX2 inhibited gene expression by enlisting PRC2 to change the repressive mark H3K27me3 [11]. The contentious findings that CBX2 had both gene-repressive and gene-active functions could not be explained by this. We found that the genes whose promoters contained the CBX2 peak were enriched in pathways related to co-regulator activity or transcription factors, indicating that CBX2’s repressive or active function may be dependent on the transcription factors (Figure 5B). This demonstrated CBX2 could empower the cell cycle indirectly. However, the CHR motif suggested that CBX2 might bind to the cell cycle-related genes in a competitive manner with the DREAM complex to lessen the repression of the targets, indicating CBX2 directly accelerated the cell cycle.

Furthermore, the p53 was dysregulated in *CBX2*^high^ tumors, destroying the p53-p21-DREAM axis or the p53-p21-RB-E2F axis, as evidenced by the higher TP53 mutation rate [36, 37]. Surprisingly, ORC1 and POLD1 were RB-E2F-controlled genes with strong correlations to *CBX2*, not DREAM targets [37] (Supplementary Figure 9H). A *CBX2* knockout prevented *CCND2* expression, and *CBX2* showed a negative correlation with *CDKN1A*, which encodes p21 (Figure 4I). This supported CBX2 could pose competition to the DREAM complex [29]. On particular mechanisms, though, more study is required.

As anticipated, *CBX2* and *CEP55* had an impact on the function states of cancer, especially the cell cycle. Additionally, *CBX2* and *CEP55* positively regulated the following processes: proliferation, metastasis, EMT, invasion, differentiation, hypoxia, wound healing, and apoptosis (Figure 6F and Supplementary Figures 12G, 14A, 14B). This suggests that *CBX2* and *CEP55* overexpression may lead to cancer stem cell-like phenotype and is supported by higher expression of *CBX2* and *CEP55* in cancer stem cells [38] (Supplementary Figure 11C). Cancer stem cells had a well-known high level of drug resistance. Lenvatinib, an immunotherapy, was more sensitive and Sorafenib, a radiation therapy, was more resistant to *CBX2*^high^ tumors (Figure 6F and Supplementary Figures 10C, 12E), indicating that proper methods should be taken into consideration for HCC therapy. Additionally, *CBX2*^high^ tumors could be treated with Voreloxin, a Topoisomerase II inhibitor that targets TOP2A, whose expression was strongly correlated with *CBX2* (Supplementary Figure 10C).

Previous research suggested that immune infiltration may affect the patient’s prognosis [39]. We found that *CBX2*^high^ or *CEP55*^high^ tumors displayed a highly complex tumor environment with both activated and suppressor cells (Supplementary Figures 13, 14). Additionally, *CBX2* was primarily found in hepatocytes or cancerous cells, where it drove the remodeling of the extracellular matrix and was significantly correlated with cancer-associated fibroblast. *CBX2* or *CEP55*’s effects on TME need to be specifically investigated.

In conclusion, using the refined regulatory network and dependable prognostic model, we verified the abnormal *CBX2* and *CEP55* in HCC. Additionally, *CBX2* may facilitate the cell cycle by directly working with co-regulators to control *CEP55* and CTNNB1 or by indirectly competing with the DREAM complex. The phenotype that resembles cancer stem cells may be enhanced by the overexpression of *CBX2* and *CEP55*. *CBX2* and *CEP55* may serve as potential drug targets and important genes for the effectiveness of immunotherapy. The triple regulatory networks predicted by the databases, the true impact of CBX2 on *CEP55* or *CTNNB1*, and the repressive or active mechanism of CBX2 are a few limitations to be aware of. Therefore, additional fundamental research is needed to investigate the direct functional mechanism.

## Supplementary Material

Supplementary Figures

Supplementary Table 1

Supplementary Table 2

Supplementary Tables 3 and 4
