# Supplementary material for: Prognostic hub gene CBX2 drives a cancer stem cell-like phenotype in HCC revealed by multi-omics and multi-cohorts
Source: Aging (Albany NY). 2023 Nov 17;15(22):12817–51. doi: 10.18632/aging.205173 (PMC10713423; doi:10.18632/aging.205173)
Supplement: Supplementary Figures [file aging-15-205173-s001.pdf]

SUPPLEMENTARY FIGURES

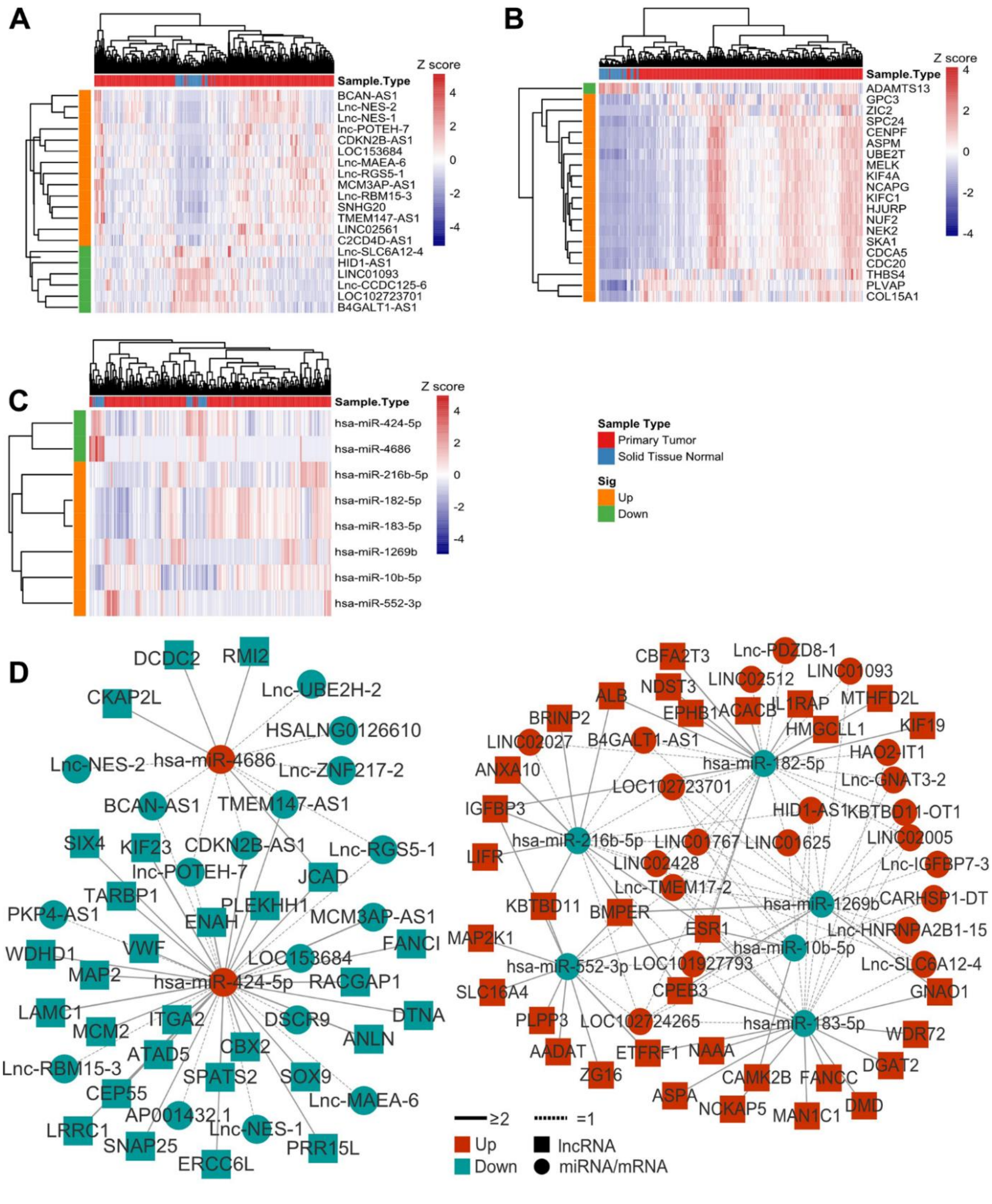

Supplementary Figure 1. Examples of differentially expressed RNAs and initial network. (**A**–**C**) Heatmap representation of top20 differentially expressed lncRNAs (**A**), mRNAs (**B**) and miRNAs (**C**). (**D**) Summary of initial regulatory network. Color indicated the differential trends Up or Down. The virtual or reality of the straight line indicated the number of databases supporting the interaction.

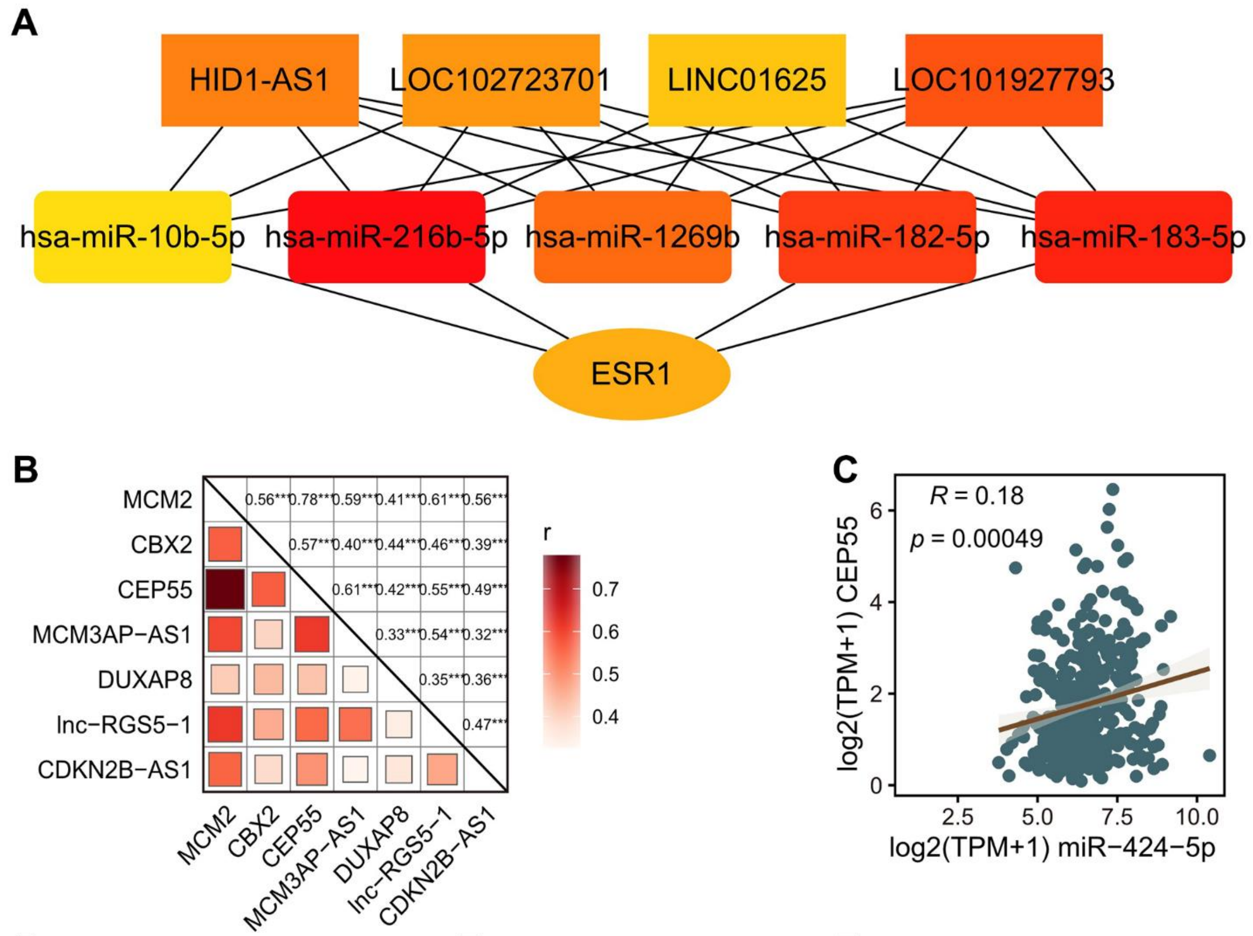

Supplementary Figure 2. Strategy-hub of refined triple regulatory networks. (**A**) Hub identified using strategy-two method from refined regulatory networks. (**B**) The Pearson's correlation of lncRNA and mRNAs in the strategy-one hub. (**C**–**F**) The Pearson's correlation between lncRNAs, mRNAs and *miR-424-5p* in the strategy-one hub.

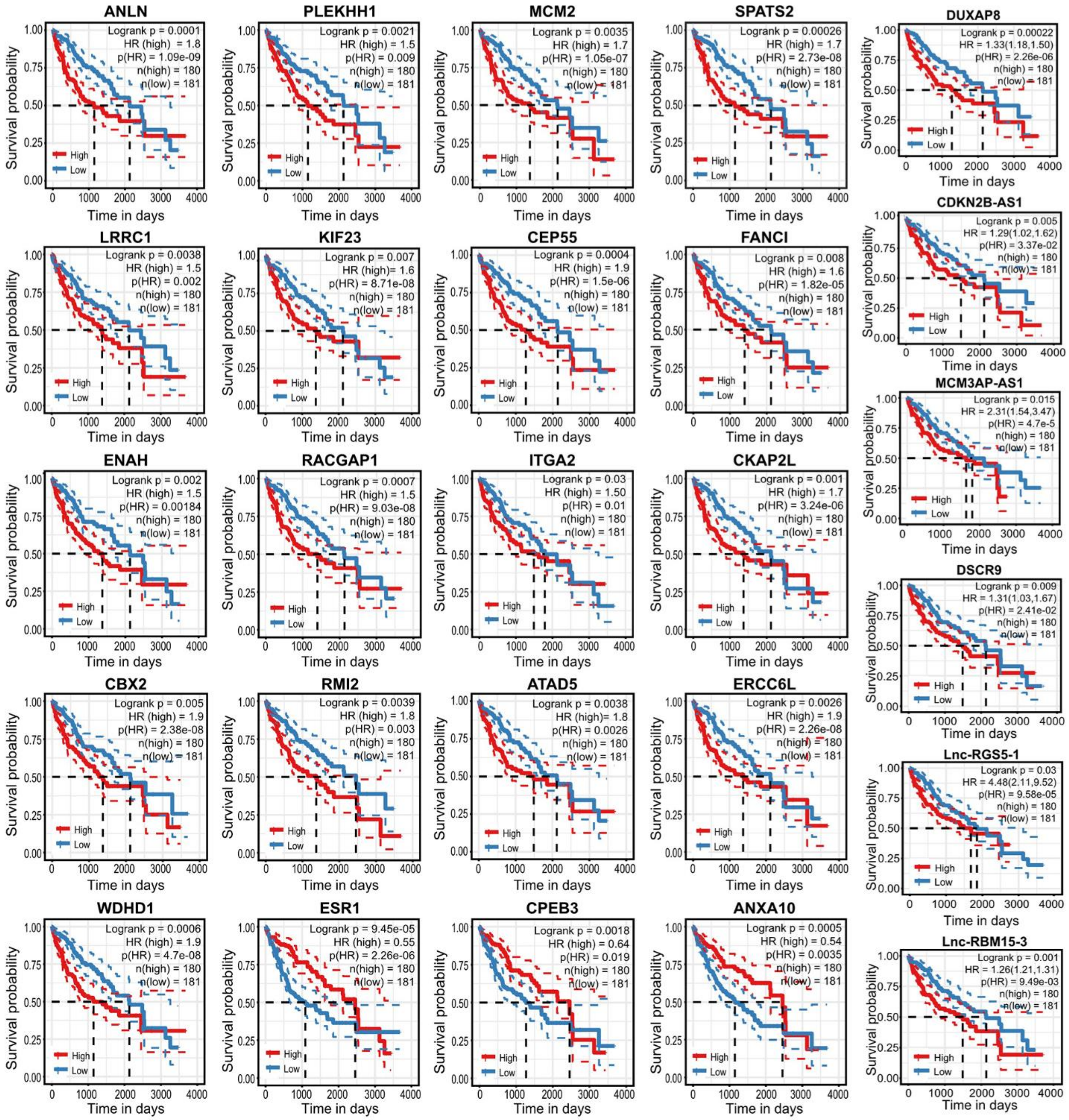

Supplementary Figure 3. Kaplan–Meier plots of genes in refined regulatory network. Kaplan–Meier plots of genes (mRNA, lncRNA, miRNA) significantly affecting patients' overall survival time in LIHC.

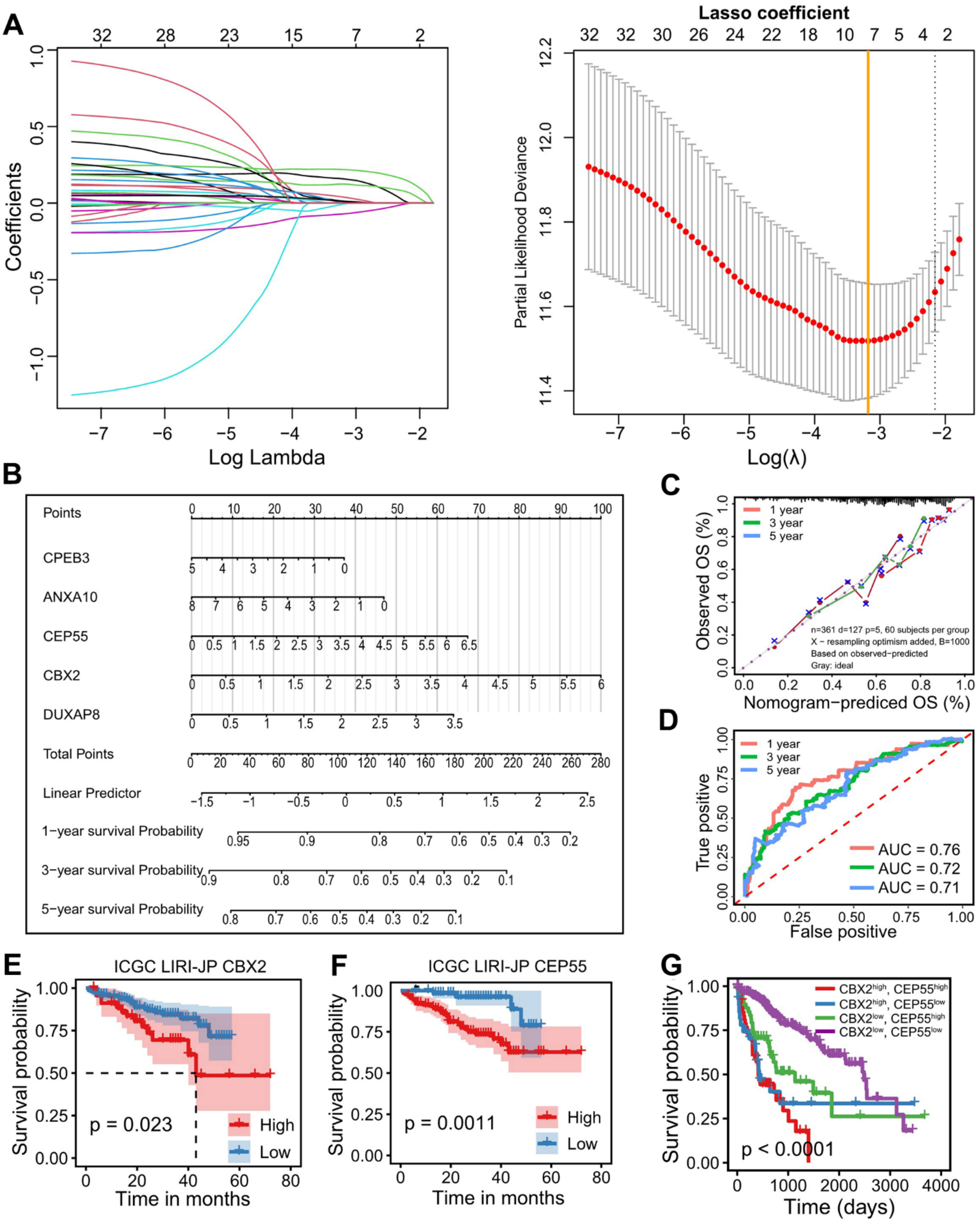

Supplementary Figure 4. Construction and validation of prognostic model. (**A**) Tenfold cross-validation for tuning parameter selection in the LASSO model and the LASSO coefficient profiles of the prognostic genes for HCC. (**B**) Nomogram to estimate the risk of HCC based on the prognostic model. (**C**) Calibration plot for prognostic nomogram in LIHC. (**D**) Time dependent ROC and AUC at 1-, 3-, and 5-year predicted using LIHC by 5-survival-gene prognostic model. (**E**, **F**) Kaplan–Meier plots of *CBX2* (**E**) and *CEP55* (**F**) in ICGC-LIRI-JP. (**G**) Kaplan–Meier plots of *CBX2* combined with *CEP55* in TCGA-LIHC.

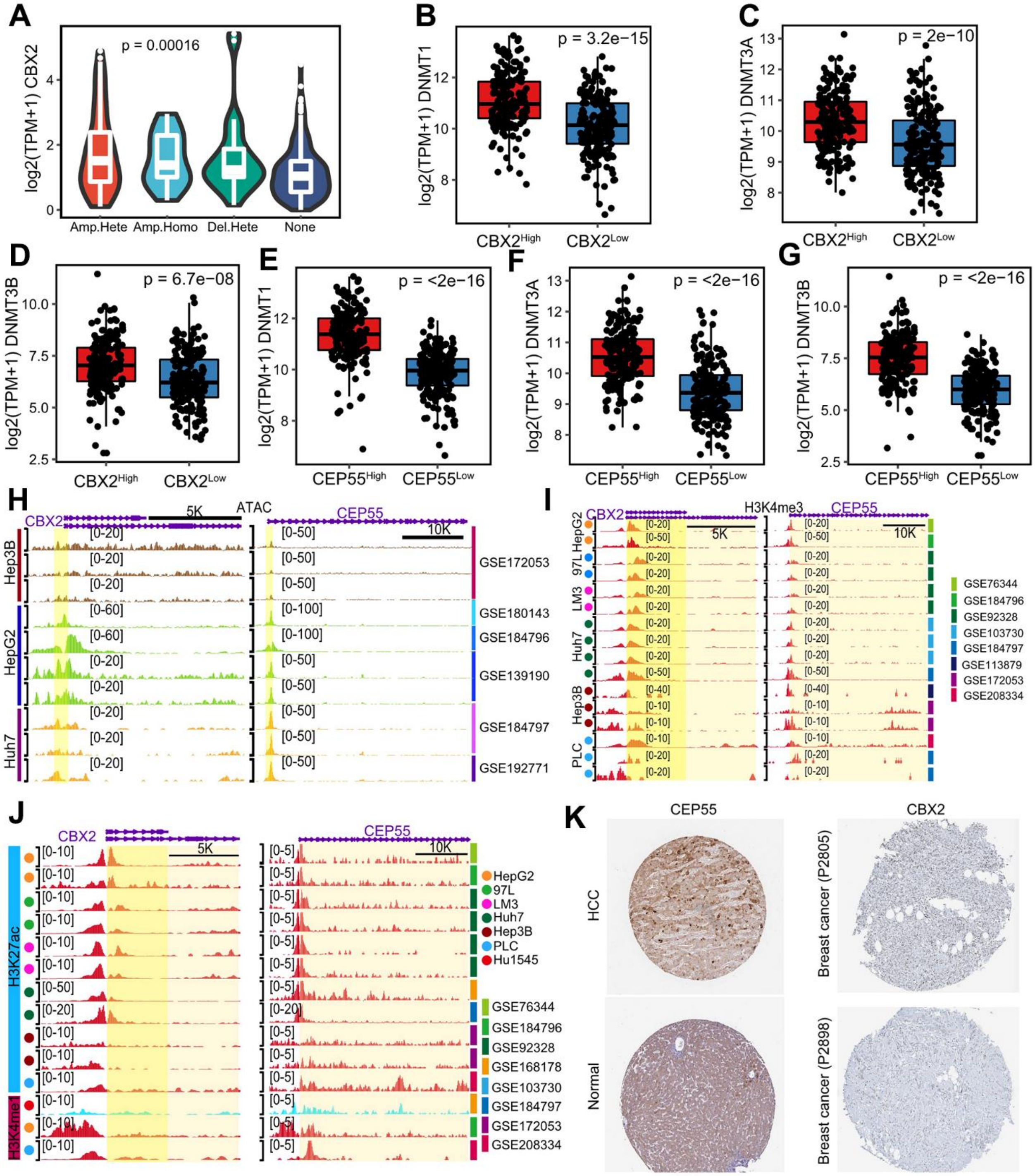

Supplementary Figure 5. Genomic and epigenomic alternations affected *CBX2* and *CEP55* expression. (**A**) Distribution of *CBX2* expression in different *CBX2* CNV type. P-value was derived from Kruskal-Wallis test. (**B**–**G**) Expression levels of DNA methyltransferases *DNMT1* (**B**, **E**), *DNMT3A* (**C**, **F**) and *DNMT3B* (**D**, **G**) in *CBX2*-stratified (**B**, **C**) and *CEP55*-stratified (**E**–**G**) tumors. (**H**–**J**) Open chromatin accessibility (**H**), H3K4me3 (**I**), H3K27ac and H3K4me1 (**J**) signals on *CBX2* and *CEP55* loci in the HCC cell lines. (**K**) Immunohistochemistry examples of *CBX2* and *CEP55* in HCC or breast cancer.

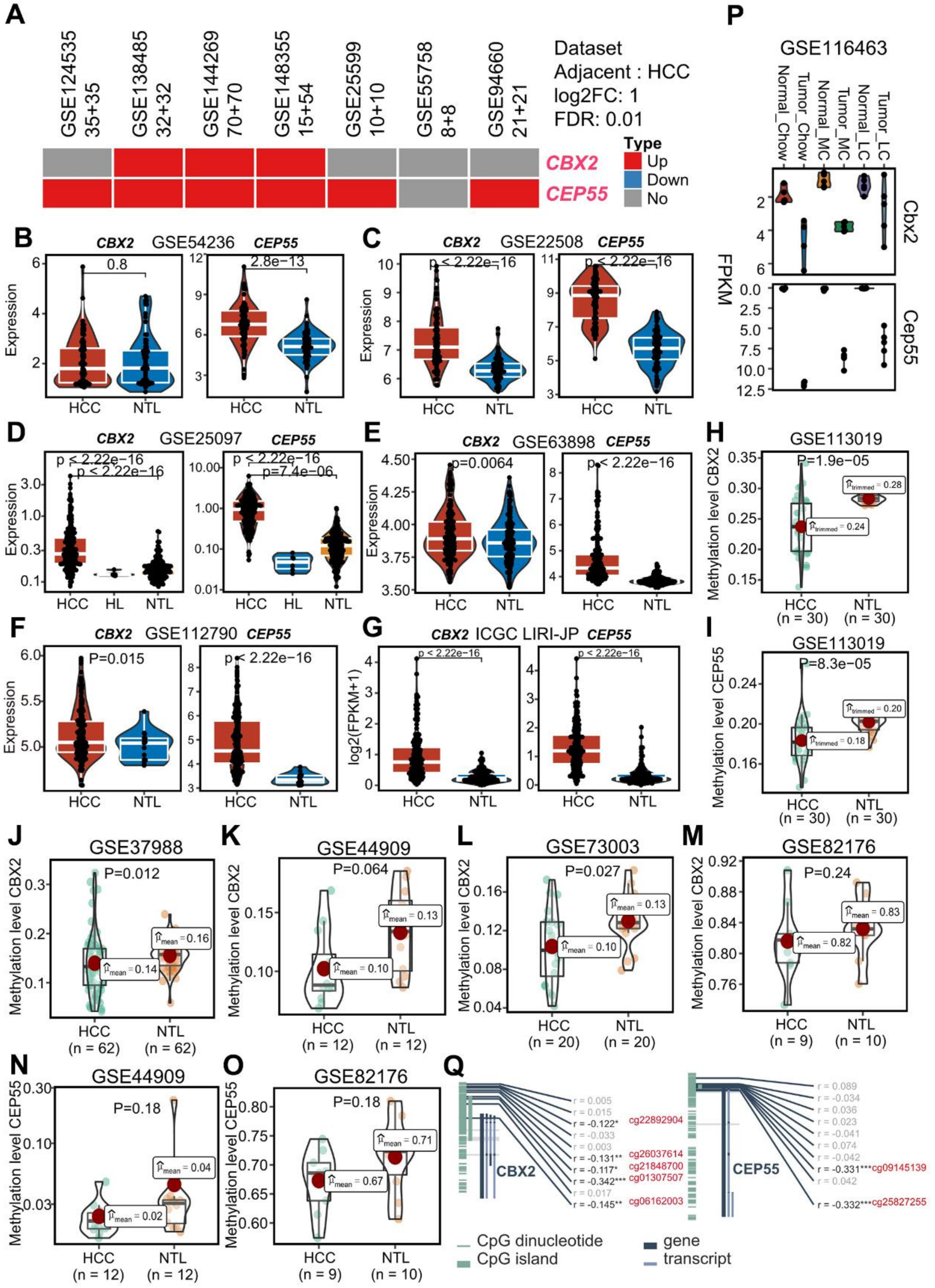

Supplementary Figure 6. Validation of *CBX2* and *CEP55* expression and methylation. (**A**) Summary of differential expression for *CBX2* and *CEP55* in 7 human HCC tissue RNA-seq datasets. Differential genes were identified by DESeq2 with log2FC 1 and FDR 0.01. (**B**–**G**) Validated expression of *CBX2* and *CEP55* in human RNA-seq datasets and array datasets. (**H**–**O**) Validated methylation level of *CBX2* and *CEP55* in 450K methylation array or EPIC methylation array datasets. (**P**) *CBX2* and *CEP55* expression in mouse tumors and normal tissues. (**Q**) MEXPRESS validated expression-associated methylation sites. P-value was calculated using the two-sided Wilcoxon test.

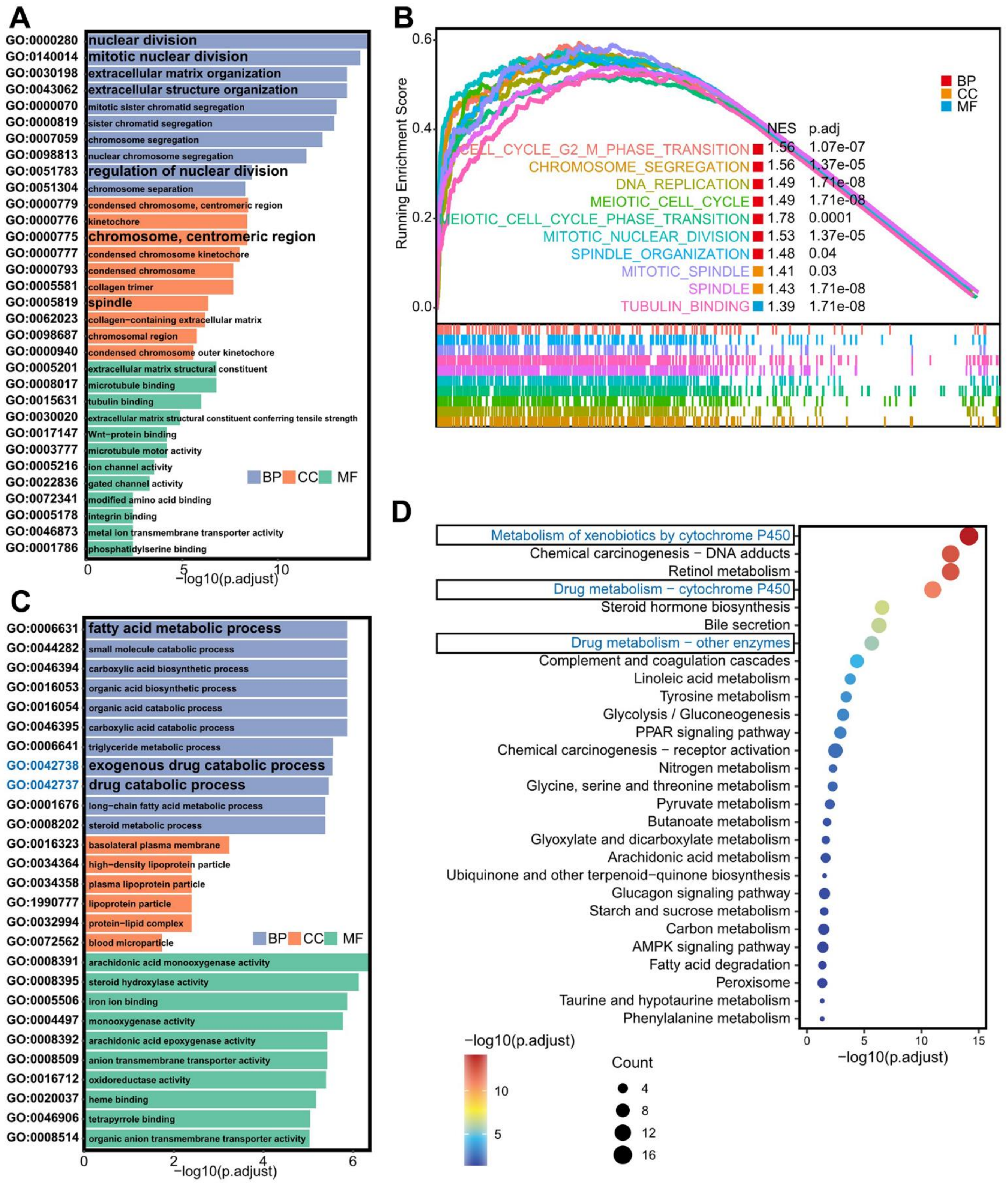

Supplementary Figure 7. Enrichment analysis for up-regulated and down-regulated genes in *CBX2*-stratified tumors. (**A**) Enriched GO terms in CBX2-related up-regulated genes. (**B**) GSEA analysis of CBX2-related GO terms. (**C**) Enriched GO terms in CBX2-related down-regulated genes. (**D**) Enriched KEGG pathways of CXB2-related down-regulated genes.

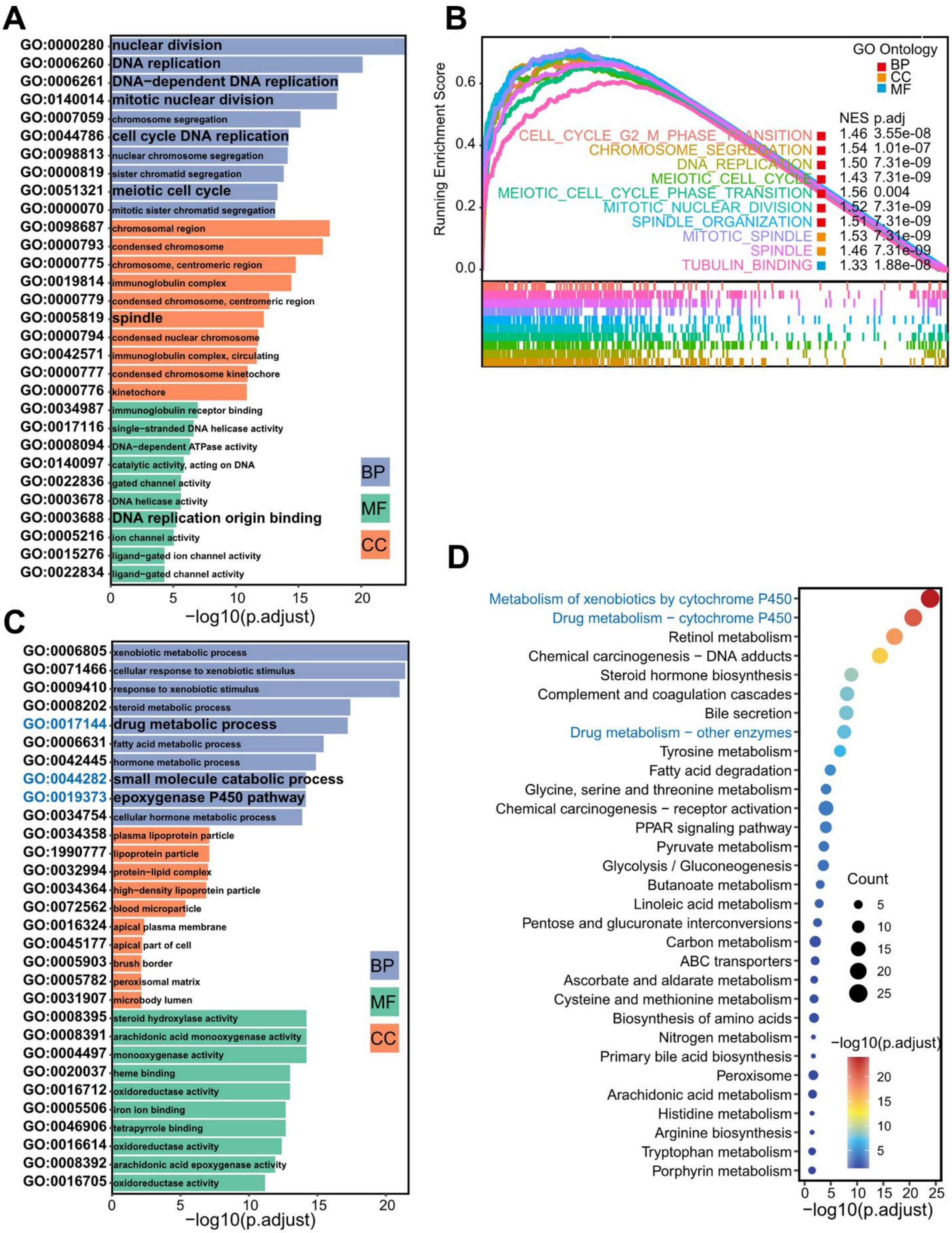

Supplementary Figure 8. Enrichment analysis for up-regulated and down-regulated genes in *CEP55*-stratified tumors. (**A**) Enriched GO terms in *CEP55*-related up-regulated genes. (**B**) GSEA analysis of *CEP55*-related GO terms. (**C**) Enriched GO terms in *CEP55*-related down-regulated genes. (**D**) Enriched KEGG pathways of *CEP55*-related down-regulated genes.

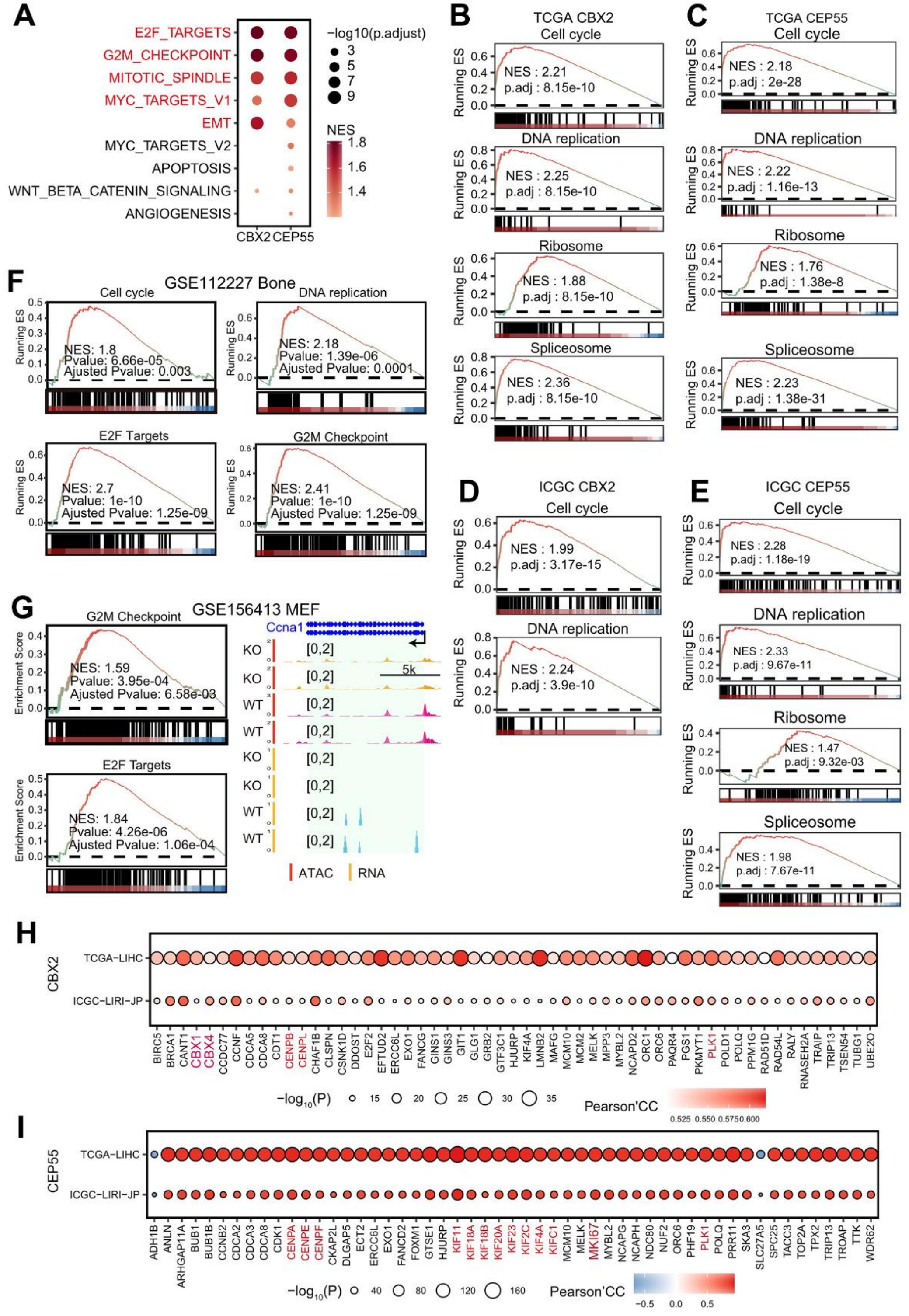

Supplementary Figure 9. *CBX2* and *CEP55* enhanced the cell cycle and cell cycle-related pathway. (**A**) GSEA analysis of *CBX2*-related and *CEP55*-related cancer hallmarks. (**B**–**E**) GSEA analysis for *CBX2*-related (**B**, **D**) and *CEP55*-related (**C**, **E**) KEGG pathways based on the Pearson Correlation Coefficient in TCGA-LIHC (**B**, **C**) and ICGC-LIRI-JP (**D**, **E**). (**F**) GSEA for *CBX2*-related KEGG pathways and cancer hallmarks using GSE112227 with *CBX2* knockdown in bone. (**G**) GSEA for *CBX2*-related cancer hallmarks using GSE156413 with *CBX2* knockdown mouse embryonic fibroblast. And the RNA and ATAC signal tracks on Ccna1 loci. (**H**, **I**) Examples of the genes that are highly correlated with *CBX2* (**H**) and *CEP55* (**I**) in TCGA-LIHC and ICGC-LIRI-JP.

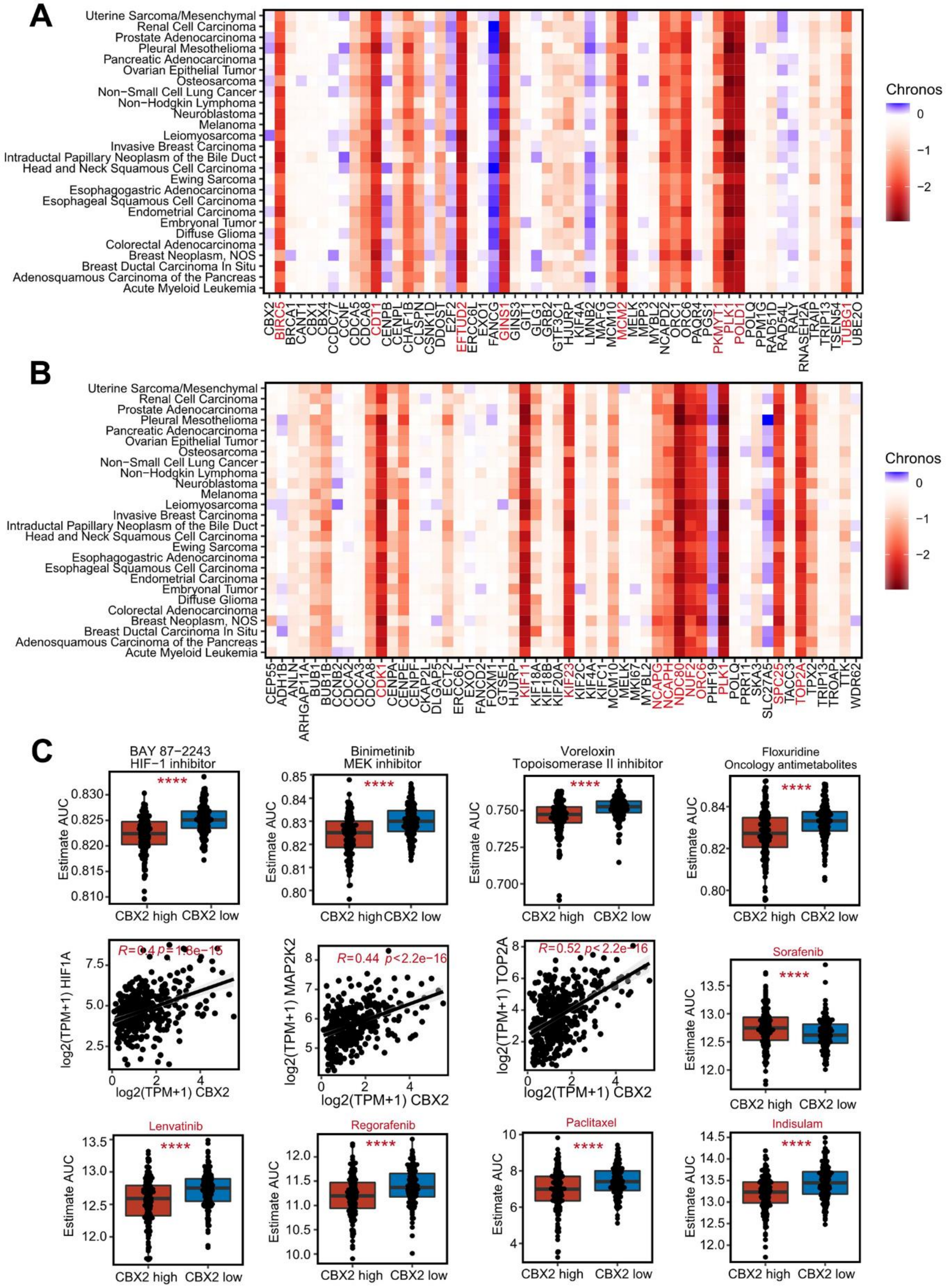

Supplementary Figure 10. Identification of the *CBX2*-sensitive drugs. (**A**, **B**) Chronos score of the genes of positively correlated with *CBX2* (**A**) and *CEP55* (**B**) shown in Supplementary Figure 9H, 9I. (**C**) Representative drugs of *CBX2*-sensitivity or resistance.

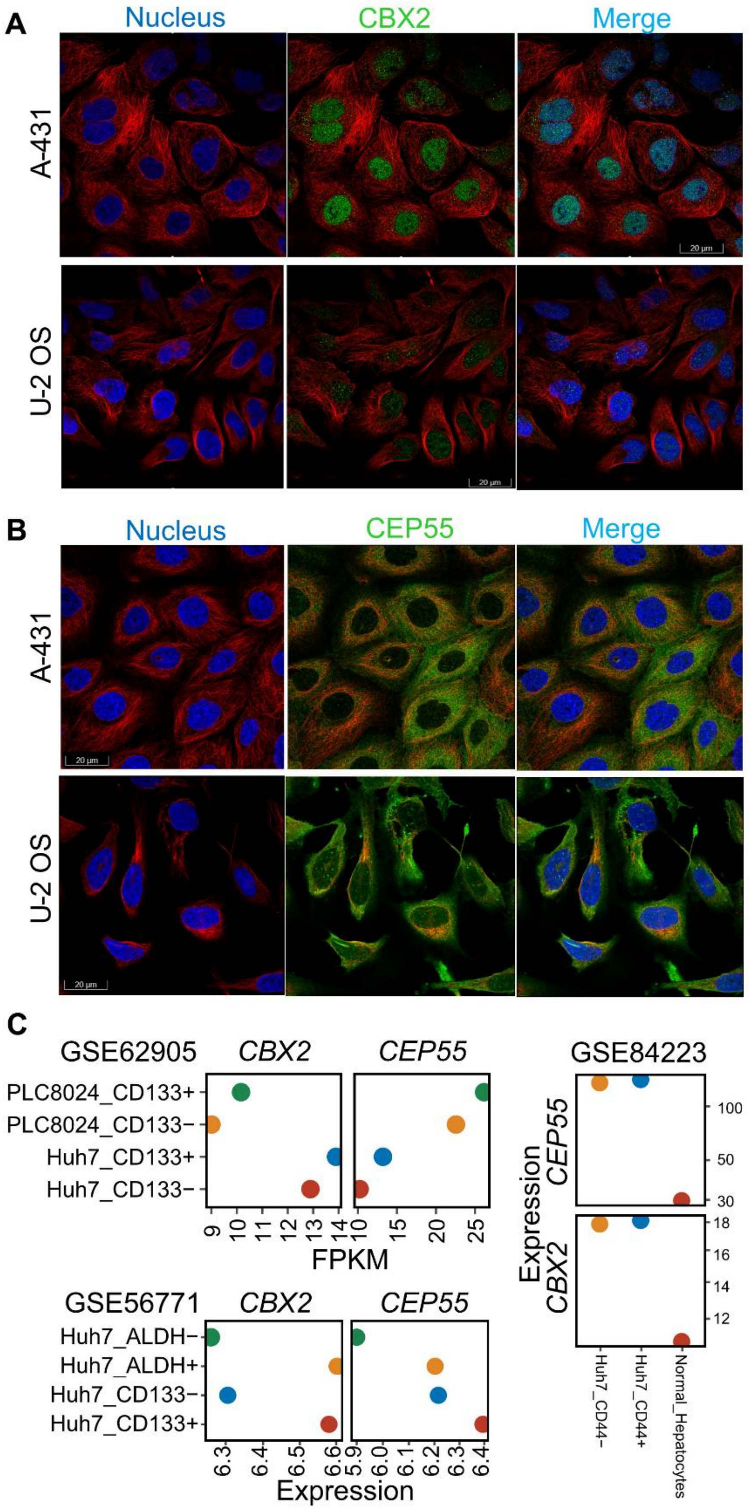

Supplementary Figure 11. Sub-cellular location and expression level in cancer stem cell of CBX2 and CEP55. (**A**, **B**) Multiplexed immunofluorescence images of CBX2 (**A**) and CEP55 (**B**) in A-431 and U-2 OS cell line. (**C**) *CBX2* and *CEP55* expression in CD133, ALDH and CD44-labelled HCC cell lines.

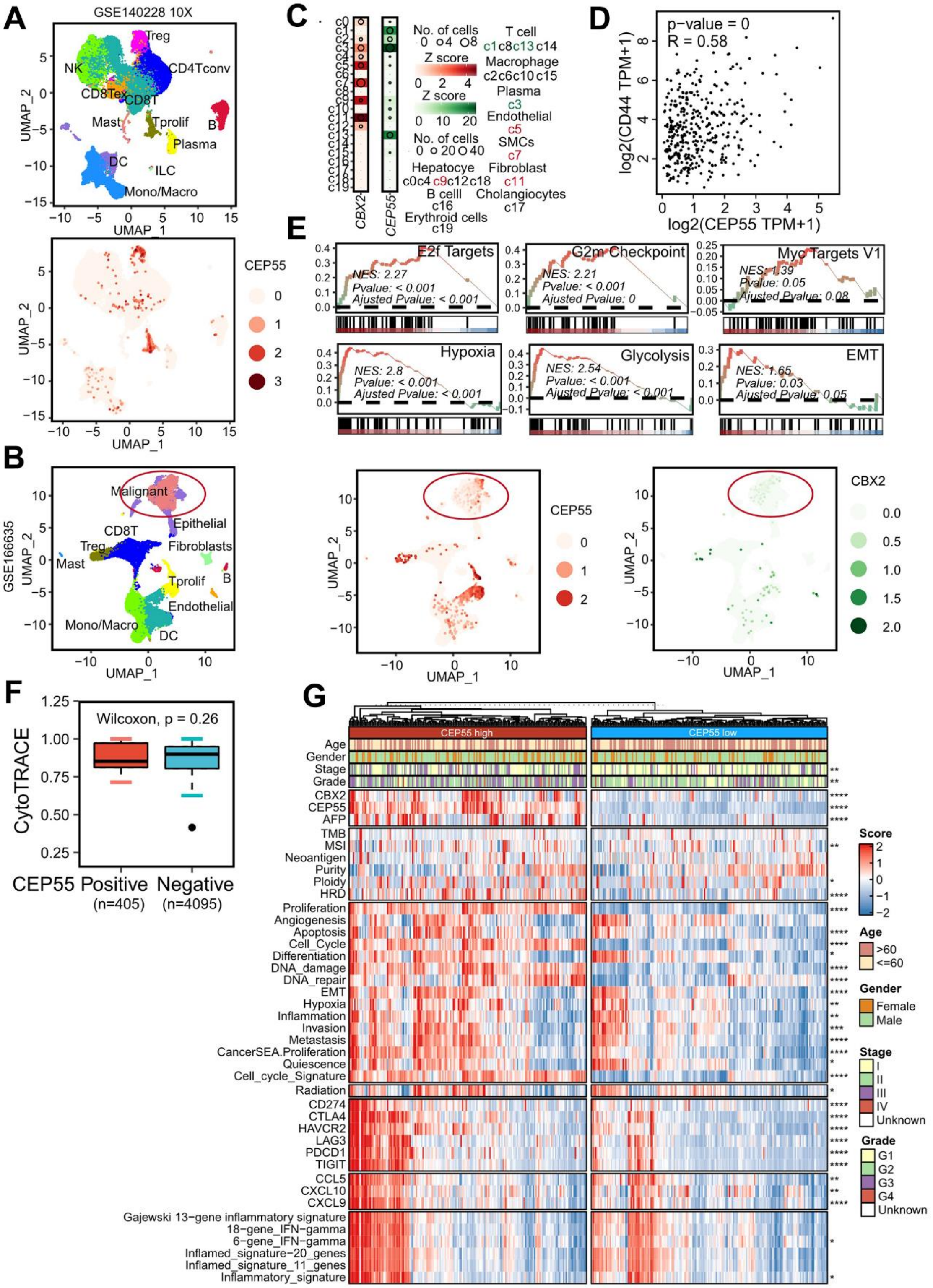

Supplementary Figure 12. Distribution of *CEP55* expression at single-cell level and the impacts on functional states and immune characteristics. (**A**–**C**) The distribution of *CEP55* and *CBX2* expression in scRNA-seq dataset GSE140228 (**A**), GSE166635 (**B**), HPA (**C**). (**D**) Pearson's correlation of CD44 and CEP55 expression level in pan-cancer. (**E**) GSEA enrichment results for hallmark gene sets with *CBX2*-positive and –negative malignant cells in GSE125449. (**F**) The distribution of CytoTRACE score in *CEP55*-positive and -negative malignant cell from GSE166635. (**G**) Heatmap representation of the main functional states, immunotherapy response predictors, representative molecular and immune characteristics in *CEP55*^{high} tumors and *CEP55*^{low} tumors.

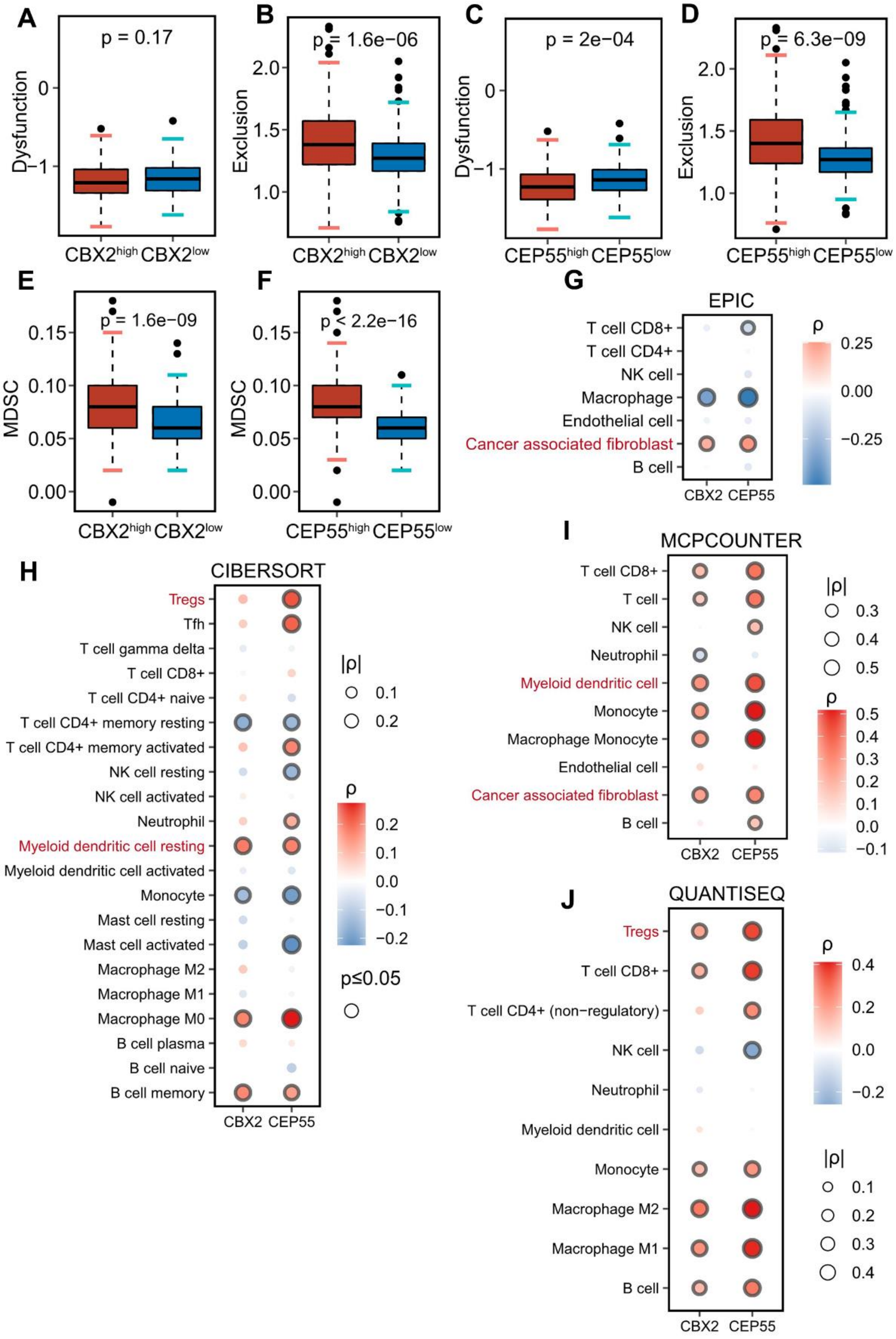

Supplementary Figure 13. Impact of *CBX2* and *CEP55* on tumor microenvironment. (**A**–**D**) Dysfunction score (**A**, **C**) and exclusion score (**B**, **D**) in *CBX2*-stratified (**A**, **B**) and *CEP55*-stratified (**C**, **D**) tumors. P value was calculated using the two-sided Wilcoxon rank sum test. (**E**, **F**) MDSC score in *CBX2*-stratified (**E**) and *CEP55*-stratified (**F**) tumors. (**G**–**J**) Spearman correlation between *CBX2* and *CEP55* and immune cell score estimated by EPIC (**G**), CIBERSORT (**H**), MCP-COUNTER (**I**) and QUANTISEQ (**J**).

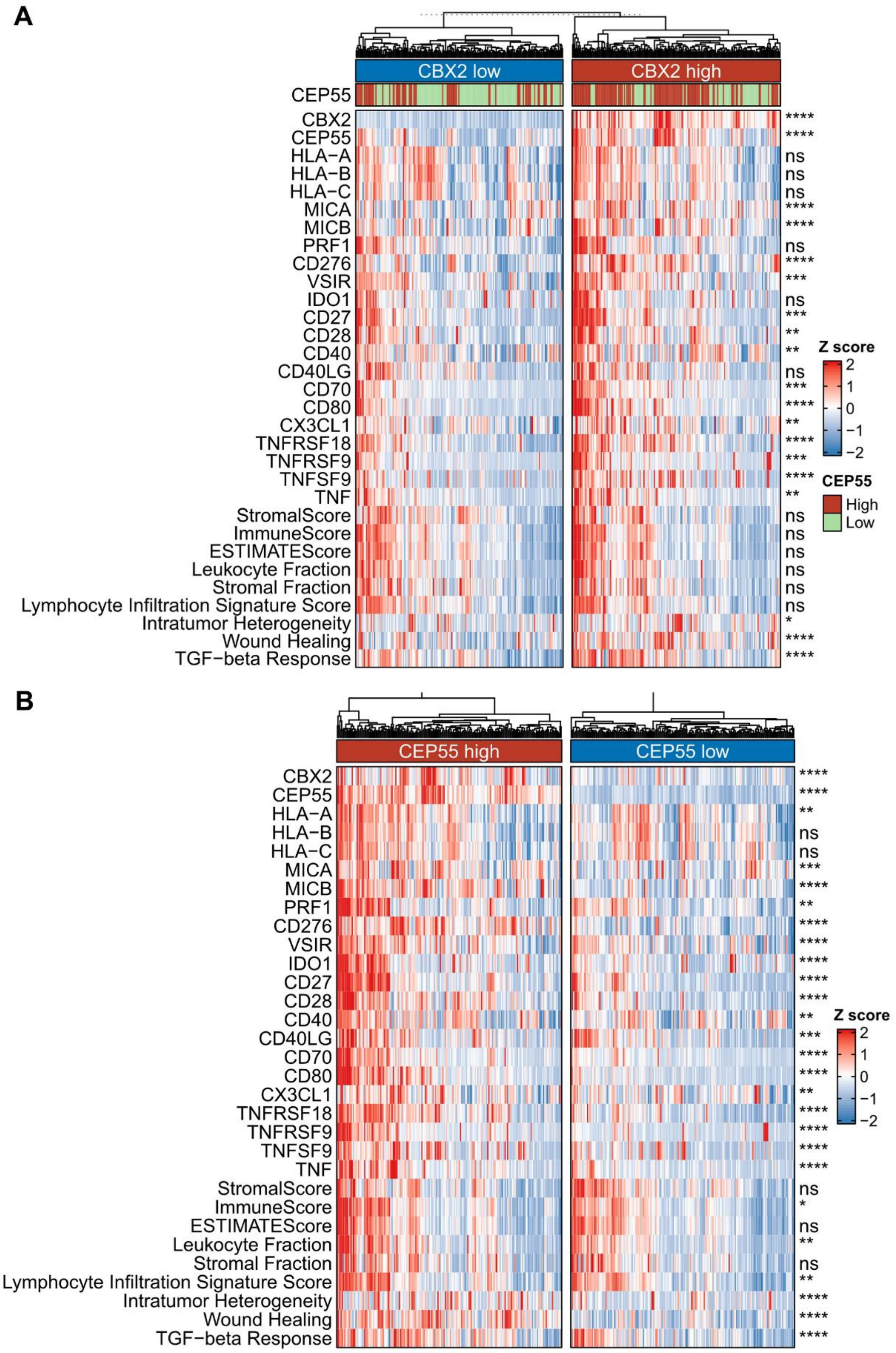

Supplementary Figure 14. Immune patterns in *CBX2*-stratified and *CEP55*-stratified tumors. (**A**, **B**) Heatmap representation of immune score, stromal score, MHC genes, immune stimulators and immune suppressors in *CBX2*-stratified (**A**) and *CEP55*-stratified (**B**) tumors. P value was calculated using the two-sided Wilcoxon rank sum test.
