# Supplementary material for: Prognostic hub gene CBX2 drives a cancer stem cell-like phenotype in HCC revealed by multi-omics and multi-cohorts
Source: Aging (Albany NY). 2023 Nov 17;15(22):12817–51. doi: 10.18632/aging.205173 (PMC10713423; doi:10.18632/aging.205173)
Supplement: Supplementary Tables 3 and 4 [file aging-15-205173-s004.pdf]

Supplementary Table 3. The deleterious mutation in *CBX2* and *CEP55*.

Gene symbol	Deleterious mutation
CBX2	0
CEP55	2

Supplementary Table 4. The effect of CNV in CBX2 and CEP55 on different survival types.

Symbol	Survival type	Logrank P value
CBX2	OS	0.07
CBX2	PFS	0.02
CBX2	DSS	0.11
CBX2	DFI	0.03
CEP55	OS	0.12
CEP55	PFS	0.37
CEP55	DSS	0.21
CEP55	DFI	0.39
